# Cryopreservation of Ram Semen: A Review on the Roles of Emerging Synthetic and Natural Additives

**DOI:** 10.3390/ani16203305

**Published:** 2026-10-20

**Authors:** Christopher Dale LeBrun, Martin Ptáček, Barbora Šedivá, Tomáš LeBrun, Lucie Langerová, Filipp Georgijevič Savvulidi

**Affiliations:** 1 Department of Animal Science, Faculty of Agrobiology, Food and Natural Resources, Czech University of Life Sciences Prague, Kamycka 129, Praha-Suchdol, 165 00 Prague, Czech Republic; ptacekm@af.czu.cz (M.P.); langerova@af.czu.cz (L.L.); savvulidi@af.czu.cz (F.G.S.); 2 Department of Chemistry, Faculty of Agrobiology, Food and Natural Resources, Czech University of Life Sciences Prague, Kamycka 129, Praha-Suchdol, 165 00 Prague, Czech Republic; buresovab@af.czu.cz; 3 Department of Management and Marketing, Faculty of Economics and Management, Czech University of Life Sciences Prague, Kamycka 129, Praha-Suchdol, 165 00 Prague, Czech Republic; lebrunt@pef.czu.cz

**Keywords:** ram semen, cryopreservation, semen extender additives, oxidative stress, post-thaw sperm quality

## Abstract

Cryopreservation techniques and protocols for ram semen, particularly those with a focus on semen extender additives, have gained popularity recently, as many scientists are looking for ways to mitigate cryodamage and improve post-thaw sperm quality. The purpose of this structured narrative review was to examine studies published in 2019–2024 on both synthetic and natural additives, to separate evidence obtained in rams from comparative evidence in other species, and to analyze current trends and potential gaps for future research. Cryopreservation is an indispensable aspect in livestock production and conservation of genes using artificial insemination. Nevertheless, the freezing and thawing processes result in DNA fragmentation, oxidative stress and low sperm motility, which are detrimental to reproductive success. Conventional extenders that contain glycerol or egg yolk have been used extensively to support cell membranes and prevent the formation of ice crystals. Nevertheless, the fertility rates obtained from frozen–thawed ram semen have been poor, which has led to the investigation of other substances. Natural additives like plant polyphenols, vitamins, fatty acids and sugars have shown great potential to decrease free radicals and enhance sperm integrity. Synthetic additives, including Trolox, MitoQ, and Mito-TEMPO, were also found to enhance the antioxidant capacity. Over the past few years, some trends have emerged, including an increased interest in plant-based antioxidants and nanoparticle-based additives. Nevertheless, several research gaps were observed, including the need for further optimization of the additive dose ratios, availability of studies on the stability of these additives when used long-term, and the compatibility of extender formulations across breeds and species. This review synthesizes recent developments in ram semen cryopreservation additives; for most additives, the evidence is presented in a single study with sperm-quality endpoints. Few studies measured in vivo fertility. It emphasizes the necessity of shifting from descriptive additive studies to mechanism-focused protocols validated across breeds to enhance the scalability and reliability of reproductive biotechnologies.

## Simple Summary

Artificial insemination using frozen ram semen is an important tool for sheep breeding and genetic conservation, but the freezing and thawing processes can cause severe damage to sperm cells. Traditional semen extenders rely heavily on egg yolk and glycerol to help protect the sperm cells, but they carry contamination risks and often lead to poor post-thaw fertility results. In order to overcome these issues, current studies are investigating novel natural and synthetic additives to assist in protecting cell membranes and reduce oxidative damage. These additives include plant extracts, vitamins, amino acids, hormones, sugars, and lipids. This review analyzes recent advancements in semen extender supplementation and highlights promising compounds. Furthermore, it identifies important research gaps such as dose standardization and long-term storage stability. Advancing these extender formulations will improve the success and reliability of reproductive technologies in sheep and other livestock species.

## 1. Introduction

Cryopreservation is the process of freezing and storing biological material at a temperature of −196 °C, and the process of cryopreserving semen has been around since the 1950s [1]. Sperm storage at −80 °C is suitable only for short periods of time, and long-term storage requires temperatures below the glass transition of the intracellular medium (around −130 °C). In practice, this is accomplished with liquid nitrogen or its vapor [2]. The cryopreservation of ram semen, however, presents many unique challenges due to the specific structure and functional sensitivity of ram spermatozoa. The freezing and thawing processes can compromise the quality of the sperm by inducing DNA fragmentation, acrosomal and membrane damage, and lipid peroxidation, which in turn can impair motility, viability and overall fertilization capacity. All of these negative effects occur due to oxidative stress, ice crystal formation, and osmotic imbalances during the freezing and thawing cycles. It is for this reason that the effectiveness of cryopreservation is dependent on the development and optimization of not only freeze–thaw protocols, but also extenders and their additives [3,4,5].

Over the years, semen extenders have been developed to help mitigate these challenges by providing sperm cells with both nutrients and a protective environment during the cryopreservation process. Many traditional extenders use cryoprotectants like glycerol and egg yolk to help prevent ice crystal formation and stabilize the cell membrane, but even with recent advancements and extender formulations, the fertility rates of ewes inseminated with frozen–thawed ram semen remain relatively low. Pregnancy rates of 50–70% are commonly reported with fresh or chilled semen. Frozen–thawed semen, on the other hand, gives rates between less than 5% and 36% when used in cervical insemination; Norway is an exception to this, where fertility rates above 60% are achieved with frozen–thawed semen without laparoscopy [6].

**Figure 1:**
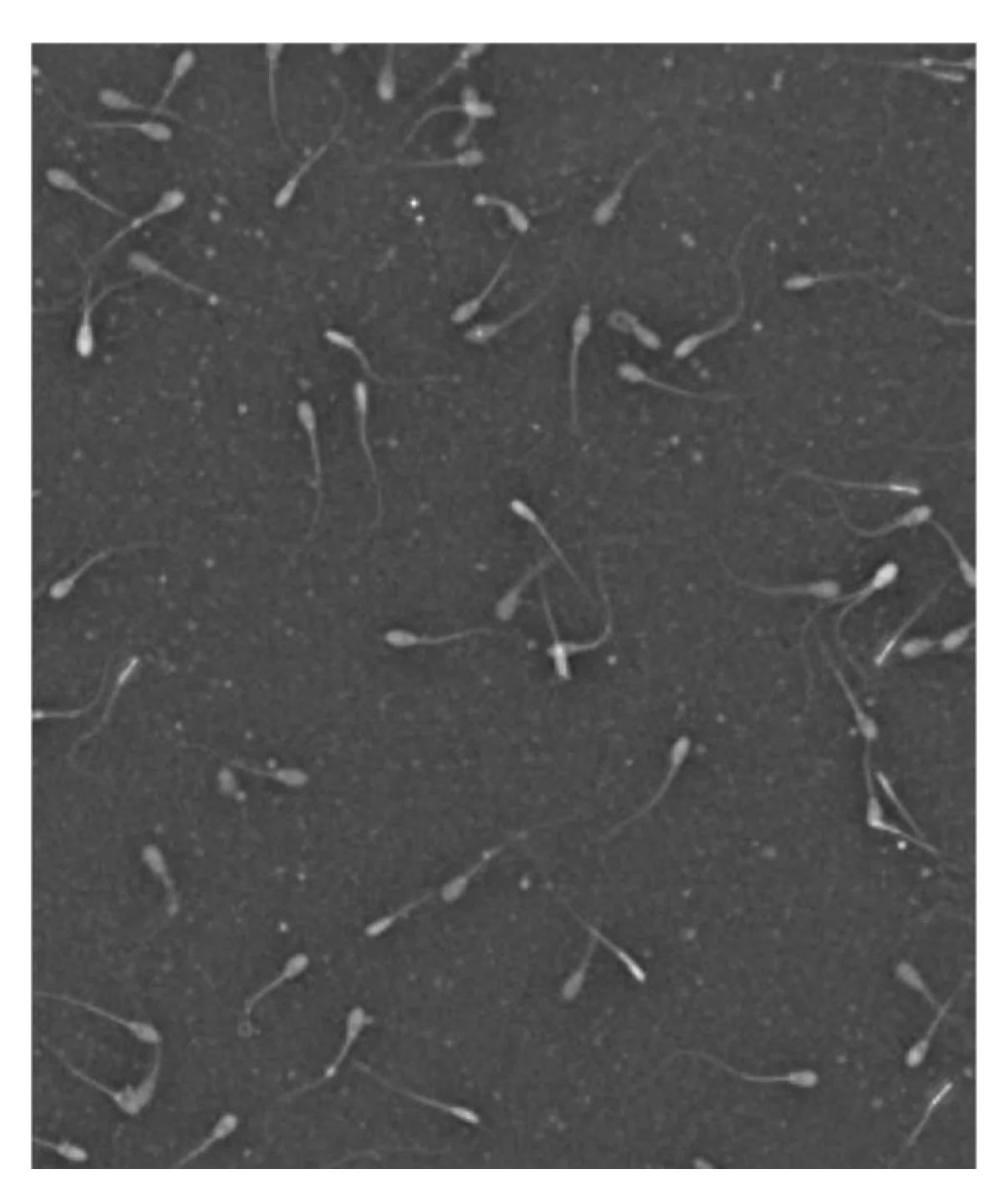
Unstained ram spermatozoa viewed using Nikon Eclipse 400 microscope (Nikon, Tokyo, Japan) with ×10 negative phase contrast optics. Image obtained by the authors during routine semen evaluation.

Ram spermatozoa exhibit typical ruminant morphology: flattened, paddle-shaped head and no unusual flagellar features (shown in Figure 1). The reason for their poor cryosurvival can be explained by their membrane composition and physiology. The ram sperm plasma membrane contains a high proportion of unsaturated phospholipids and little cholesterol, which lowers its resistance to both cooling and freezing and makes it susceptible to lipid peroxidation [3]. The loss of membrane cholesterol induced by cooling also triggers capacitation-like changes (cryocapacitation) in frozen–thawed sperm, which can impair their fertilizing ability [7]. Furthermore, the ewe cervix is narrow and sinuous, so frozen–thawed sperm deposited in the cervix are poorly transported. As a result, acceptable fertility, with frozen–thawed doses, generally requires laparoscopic intrauterine insemination [6].

As a result, research has started to focus on incorporating novel synthetic and natural additives, such as antioxidants, vitamins, fatty acids, and other bioactive compounds [8,9,10,11]. Some studies have explored adding antifreeze proteins (AFPs) to supplement semen extender formulations. AFPs are ice-binding molecules that help mitigate cryoinjury through the inhibition of ice crystal formation by stabilizing cell membranes through direct lipid interactions. When added to ram sperm extenders, low concentrations have shown improved plasma membrane integrity and specific kinematic parameters. The results are inconsistent across AFP types, doses, and extenders [12,13].

The choice of extender can also influence the kinetics of sperm motility, which are commonly used as indicators of fertilizing potential. Studies have indicated that different extenders can yield varying results on sperm motility parameters, such as progressive motility and velocity [14,15]^†^. For instance, extenders enriched with specific antioxidants or sugars have been shown to enhance motility characteristics, which are an important prerequisite for fertilization [16,17,18]. Furthermore, the interaction between the extender components and the spermatozoa can significantly affect the overall success of artificial insemination programs, emphasizing the need for careful selection and optimization of extender formulations. The aim of this structured narrative review was to examine studies published in 2019–2024 on synthetic and natural additives for ram semen extenders, to separate evidence obtained in rams from comparative evidence in other species, and to identify gaps for future research. It emphasizes the necessity of shifting from descriptive additive studies to mechanism-focused protocols validated across breeds to enhance the scalability and reliability of reproductive biotechnologies.

## 2. Materials and Methods

### 2.1. Review and Design Reporting

**Figure 2:**
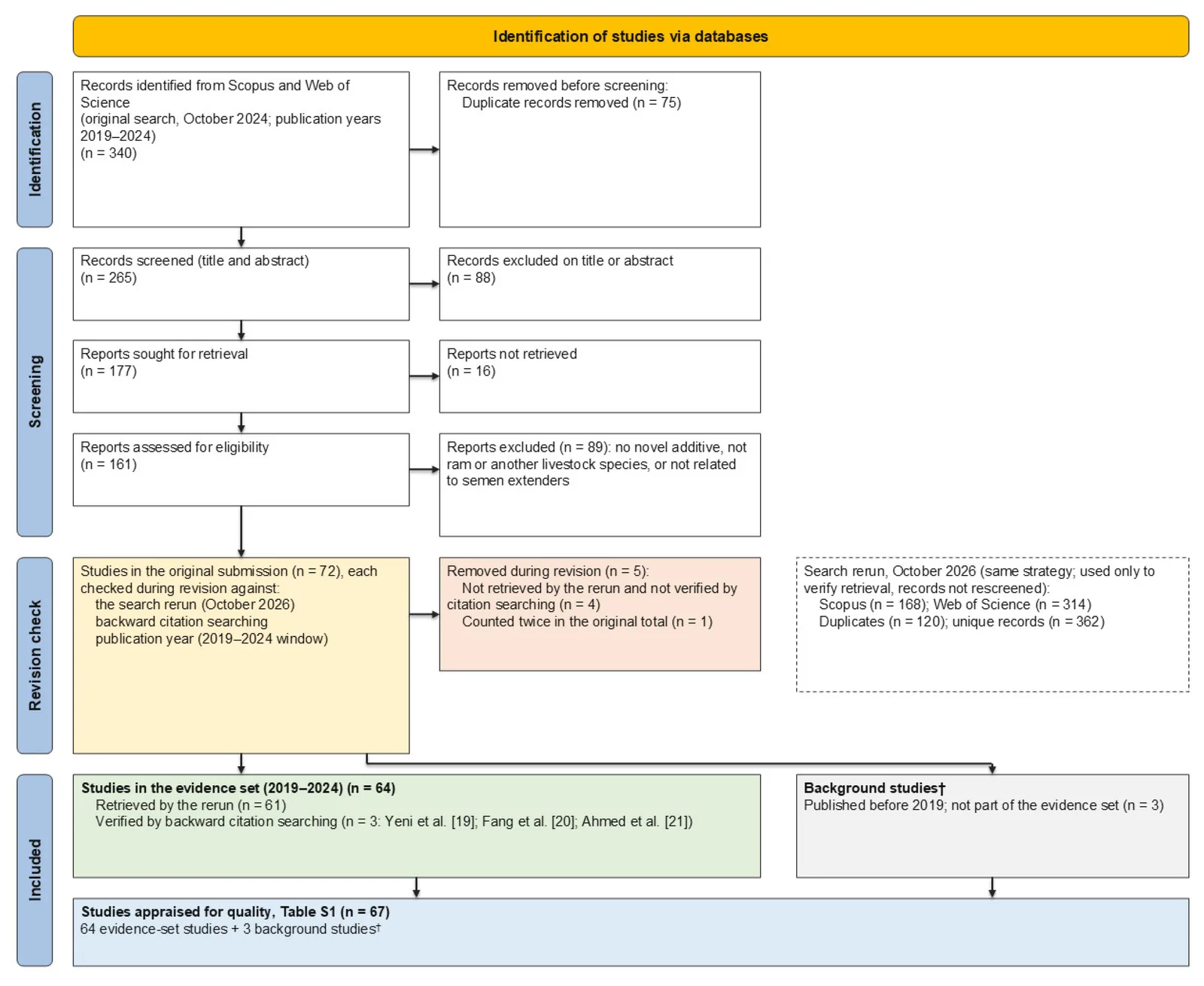
Flow of records through literature identification, screening, and selection for the structured narrative review (adapted from the PRISMA 2020 flow diagram). The diagram reflects the original search (October 2024). The October 2026 rerun was used only to verify that the included studies are retrieved by the search strategy, and its records were not rescreened. ^†^ Studies published before 2019 that were used for background only and are not part of the evidence set [19,20,21].

This is a structured narrative review. A systematic or meta-analytic synthesis was not attempted because the studies differ in species, sperm source, extender, storage model, concentration units and endpoints. In order to make the process transparent, the search strategy, eligibility criteria, study selection, and data extraction steps are described below. Figure 2 summarizes the flow of records through identification, screening, and finally selection. No review protocol was registered.

### 2.2. Information Sources and Search Strategy

The initial searches were conducted in Scopus and Web of Science in October 2024. The complete strategies used were: Scopus—TITLE-ABS-KEY ((ram AND semen AND cryopreservation) AND (alternative* OR supplement* OR antioxidant* OR natural)), limited to publication years 2019 to 2024 and to the document types article, review, and conference paper; Web of Science Core Collection—TS = ((ram AND semen AND cryopreservation) AND (alternative* OR supplement* OR antioxidant* OR natural)), refined to the same years and to the document types article, review article and proceedings paper. The Boolean operators AND and OR and the truncation symbol (*) were used. NOT was not used. The term “synthetic” was not part of the search strategy, as synthetic compounds were retrieved through the terms supplement*, antioxidant*, and alternative*, each of which describes the additive’s role rather than origin. The searches were rerun in October 2026 using the recovered original Boolean strategy. The rerun exports contained 482 records: 168 from Scopus and 314 from Web of Science. Matching duplicate records by normalized titles identified 120 duplicates, which left 362 unique records. The rerun was used to confirm that the search strategy is reproducible. The records were not rescreened. During revision, a targeted backward-citation check was conducted to investigate ram studies cited in the review that were absent from the rerun exports; citation links were verified against reference lists of retrieved publications. This check confirmed routes to three additional reports: Yeni et al. [19], Fang et al. [20], and Ahmed et al. [21]. These reports were recorded separately from database-retrieved records, and identification through citation searching did not alter eligibility criteria. Four studies that were cited in the original submission could not be retrieved through the rerun search and could not be verified through the citation search were removed from the evidence set.

### 2.3. Eligibility Criteria

Studies were eligible for full-text screening if they were primary experimental studies in which a natural or synthetic compound or material was added to a semen extender or diluent for ram spermatozoa and compared at least one sperm-quality, biochemical, functional or fertility endpoint to a control group. Reviews and book chapters were not used as evidence, but they were used for background. The evidence set was defined by the 2019–2024 time window. Earlier publications identified through citation searching were included to provide historical and methodological context and are marked with † in the text and tables. They were not used to support conclusions on efficacy. Studies in buck, bull, boar, stag, and human were included as comparative evidence and are not used to support conclusions about rams; they were analyzed separately and clearly labeled as “comparative evidence” throughout.

### 2.4. Study Selection and Data Extraction

After removal of duplicates, titles, abstracts and full texts were screened for inclusion and assessed by a single reviewer and checked by a second reviewer on a random sample of 30% of the records. Disagreements were resolved by discussion and then consensus. After exclusion screening, as shown in Figure 2, a total of 64 studies in the evidence set (plus 3 earlier background studies) were used to perform this review. Data were extracted by one author using a pre-specified form with the following fields: species; sperm source (ejaculated or epididymal); storage model; extender and supplements; concentration and unit as reported; endpoints; and effect relative to the control. Multiple reports of the same experiment were not found. We acknowledge that screening and data extraction by a single reviewer, with independent checking of only a subset of records, is a limitation of this review.

### 2.5. Storage Models and Endpoints

Four models were distinguished: chilled storage (liquid storage at about 0–5 °C); transport/ambient storage (around 15–22 °C); cryopreservation (freezing to −196 °C and thawing); and oxidative-stress challenge (fresh or thawed sperm incubated with pro-oxidants, usually around 37 °C). Endpoints were assigned based on the following criteria: E1, sperm-quality variables (motility, kinematics, viability, membrane, acrosome and mitochondrial integrity, DNA integrity); E2, oxidative and biochemical markers (reactive oxygen species (ROS), malondialdehyde (MDA), antioxidant capacity and enzymes); E3, in vitro functional tests (cervical-mucus penetration, oocyte binding, in vitro fertilization); E4, in vivo fertility (pregnancy or lambing rate). E1 and E2 are surrogate endpoints and are not equated with fertility.

### 2.6. Appraisal of Study Quality

Each included study was appraised with a checklist of seven items, which were adapted from the SYRCLE risk-of-bias tool [22] and the ARRIVE 2.0 guidelines [23]: (1) blind assessment or random allocation of aliquots; (2) number of males and ejaculates reported and pooling stated; (3) independent replicates and statistical unit defined; (4) statistical analysis appropriate to the design; (5) sperm assessment described (staining, number of cells, computer-assisted sperm analysis); (6) additive (supplement) concentration, unit and extender fully reported; (7) control group present. Each item was scored yes, no, or unclear. Studies with six or seven “yes” were rated low concern, four or five moderate concern, and three or fewer high concern (see Table S1). Out of the 67 studies appraised, 18 were rated low concern, 36 moderate concern, and 13 high concern. The most frequent shortcomings were that random allocation or blinded assessment was not reported (61 studies); independent replicates or the statistical unit were not defined (31 studies); and that the additive concentration, unit or extender was incomplete (28 studies). The appraisal did not lead to the exclusion of any study, but rather determined how the findings are worded. It should be noted that the checklist was adapted for this review and has not been validated. As such, it reflects the completeness of reporting as much as the conduct of the studies, and ratings therefore indicate reporting and methodological concern rather than invalidity of a study.

**Figure 3:**
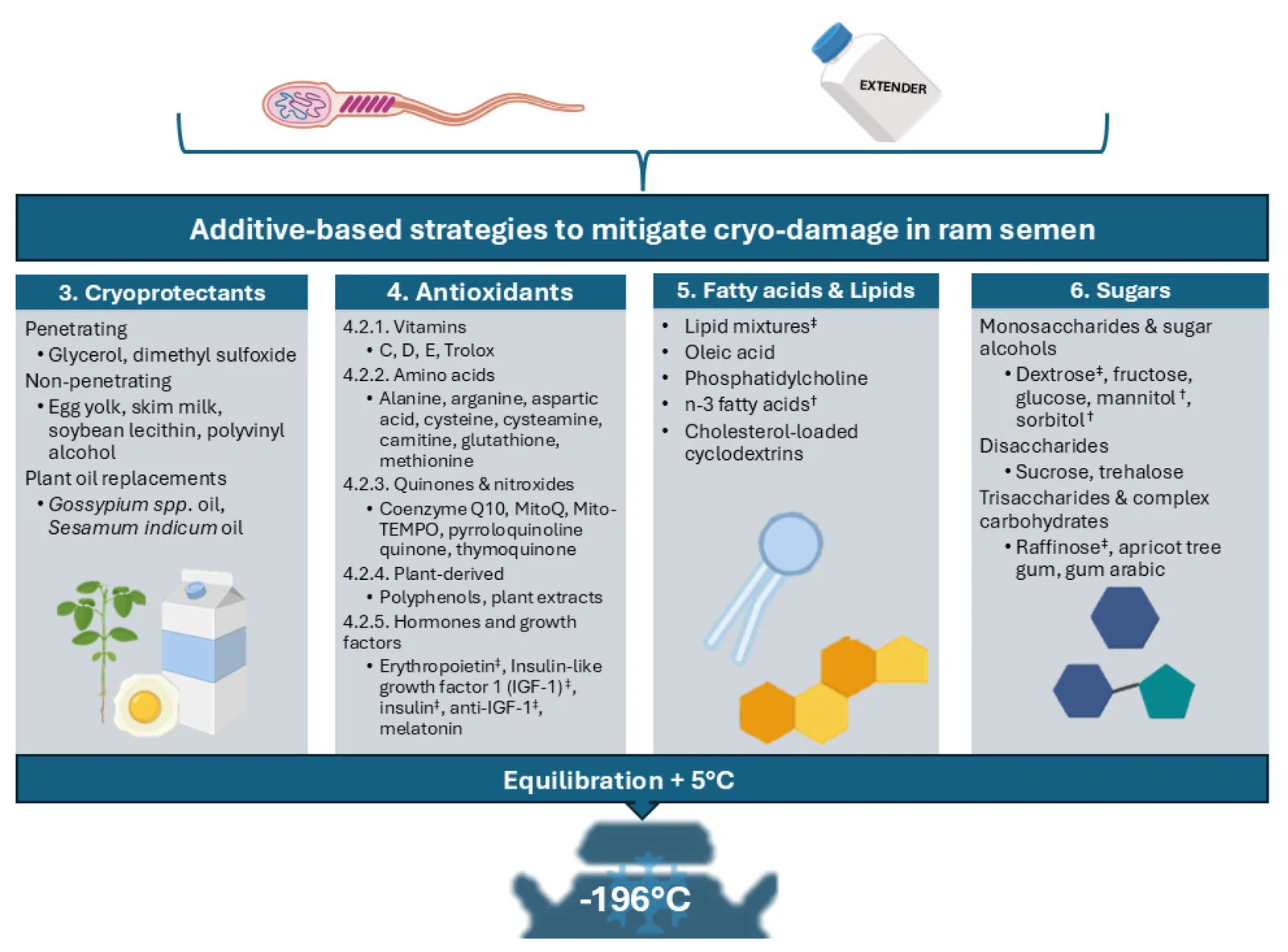
Additive-based strategies to mitigate cryo-damage in ram semen. Comparative studies are marked with ^‡^. Background studies before 2019 are marked with ^†^.

Figure 3 provides a quick reference of all the additives discussed and where to find them in this review.

Schematic overview of additive classes incorporated during ram semen cryopreservation. Additives are organized into cryoprotectants, antioxidants, saccharides, and fatty acids and lipids, reflecting the structure of the present review. Numbers adjacent to each group indicate the corresponding sections in which these additives are discussed.

## 3. Cryoprotectants

The effectiveness of cryopreservation largely depends on the choice of cryoprotectants, which can be classified into permeable and non-permeable diluents. Understanding the roles and mechanisms of these cryoprotectants is essential for optimizing semen quality post-thaw, which may support fertility. Dehydration and ice crystal formation are two of the main concerns during freeze–thaw processes [24,25]^†^, and the role of cryoprotective agents is to decrease the rate at which both occur and still allow dehydration to continue at low subzero temperatures [26]^†^. This provides the cells with additional time to respond during the formation of extracellular ice, which consequently reduces the formation of intracellular ice [27]. Moreover, cryoprotectants stabilize proteins and membranes according to the “preferential exclusion theory.” This theory suggests that water molecules preferentially interact with proteins and membranes rather than cryoprotectants. By being excluded from direct interaction, cryoprotectants assist in preserving the native structure of proteins and membranes [26,27]^†^.

The choice between permeable and non-permeable cryoprotectants is influenced by several factors, including the initial quality of the semen, the freezing protocol employed, and the specific requirements of the breeding program. For example, studies have indicated that the effectiveness of non-permeable cryoprotectants can vary significantly based on the initial quality of the sperm sample, with lower quality samples showing reduced cryoresistance when preserved without permeable agents [28,29]. This highlights the importance of tailoring cryopreservation protocols to the specific characteristics of the semen being preserved.

Permeable, or penetrating, cryoprotectants, such as dimethyl sulfoxide (DMSO), glycerol, and ethylene glycol, are widely used in the cryopreservation of ram semen due to their ability to penetrate the sperm cell membrane [28,30]. Mehta et al. [31] found favorable results with the use of 6% glycerol but had the highest percentage of post-thaw viable sperm with 2% ethylene glycol, while Najafi et al. [32]^†^ concluded that both glycerol and ethylene glycol had similar results at different concentrations (3% and 5%), with DMSO providing the least amount of viable sperm. A recent study by Hadi et al. [33] showed that an extender using 2% glycerol supplemented with polyvinyl alcohol (PVA) significantly improved post-thaw viability, motility, acrosome integrity and longevity of epididymal ram sperm. It should be noted that due to the potential toxicity of permeable cryoprotective agents at high concentrations, careful optimization, for example, altering the timing of the addition of glycerol just before freezing, must be implemented [34,35]^†^.

In contrast, non-permeable cryoprotectants, such as egg yolk, skimmed milk, and soybean lecithin, do not enter the sperm cells but instead provide protection by stabilizing the membrane, preventing ice crystal formation outside the cells, and providing essential nutrients [36].

Egg yolk has been widely utilized in semen cryopreservation due to its rich composition of lipids and proteins, which help protect spermatozoa from cold shock and osmotic stress during freezing. It acts as a membrane stabilizer, reducing the risk of damage to the sperm’s plasma and acrosomal membranes, which is important for maintaining motility and fertilization ability post-thaw. The cholesterol and lecithin content have been shown to play a key role in cryoprotection [28]. However, egg yolk is poorly standardized, as its composition varies with hen breed, diet, and age and between batches, making extenders difficult to reproduce and compare between laboratories and studies [37]^†^. Swelum et al. [38] tested the effects of adding mixed chicken and quail egg yolks, since chicken egg yolks can improve vitality, motility, and the integrity of both the plasma membrane and DNA, and quail egg yolks can lower the number of sperm abnormalities and improve sperm kinetic parameters. Unfortunately, their results showed that the mix did not provide any benefit or improvement. Nonetheless, concerns regarding the potential transmission of pathogens through egg yolk have prompted researchers to explore alternative cryoprotectants, such as skimmed milk and soybean lecithin [39]^†^.

Skimmed milk has been shown to be an effective extender for ram semen, providing a suitable environment for sperm preservation. Its casein and whey proteins contribute to the antioxidant capacity of the extender, thereby mitigating oxidative stress during the freezing process [40]^†^. Studies have indicated that skimmed milk-based extenders yield satisfactory results in preserving ram semen quality, often comparable to traditional egg yolk extenders [34]^†^.

The utilization of soybean lecithin as a component in ram semen extenders has garnered significant attention in recent years due to its potential benefits in cryopreservation and preservation of sperm quality. For instance, Gogol et al. [8] reported that the lambing rates in ewes inseminated with semen frozen in a milk–soybean lecithin extender were higher than those using a Tris–egg yolk extender, although the difference was not statistically significant. Additionally, Masoudi et al. [41]^†^ highlighted that soybean lecithin has beneficial effects in protecting ram sperm against cryoinjuries, particularly when combined with specific dietary supplements such as fish oil. Mohamed et al. [42] noted that extenders containing soybean lecithin exhibited superior characteristics in terms of motility and acrosome integrity compared to those based solely on egg yolk. This aligns with findings from Crespilho et al. [43]^†^, who reported that the use of lecithin-based extenders resulted in improved fertilizing potential in bovine semen; this is comparative evidence. However, soybean lecithin has also been reported to interfere with mitochondrial function in frozen–thawed ram sperm [39]^†^. Such comparative studies are essential for establishing the practical applications of soybean lecithin in reproductive technologies.

A recent novel approach tested the use of various seed extracts as a replacement for egg yolk in extenders. *Gossypium* spp. and *Sesamum indicum* are readily available in many African countries and naturally contain lecithin and unsaturated fatty acids. Akourki et al. [44] found that a range of 2.5% to 10% of *Gossypium* spp. oil or 1.25% to 10% of *Sesamum indicum* oil can be used to replace egg yolk in a Tris–egg-yolk-based extender for chilled storage, but more studies should be done to confirm the suitability of this replacement for frozen storage.

## 4. Antioxidants

Since the oxidative stress caused by the freeze–thaw process can damage spermatozoa functionality and viability, the use of antioxidants in semen extenders has gained popularity in recent years, especially with cryopreservation in mind. Oxidative stress is characterized by an imbalance between free radicals, mainly represented by ROS, and antioxidant defenses. Sperm cells have a high number of polyunsaturated fatty acids (PUFAs), which make them susceptible to lipid peroxidation due to the activity of ROS created during the cryopreservation process [7]. ROS have been shown to impair motility, reduce mitochondrial activity, damage DNA, and activate apoptotic pathways. Sperm can resist and react to this stress using antioxidants found in both the sperm cell and seminal plasma; however, their antioxidant capacity during cryopreservation is insufficient to combat the high number of ROS produced. Diluting and cooling both contribute to a diminished performance of endogenous antioxidants, so in an effort to mitigate these effects, research has explored adding antioxidants and/or their substrates to extenders, which has been promising, but further research is needed to optimize their concentrations and combinations [7,45,46].

### 4.1. Enzymatic Antioxidants

Enzymatic antioxidants are generally endogenous, meaning that they naturally occur in either the sperm cell or seminal plasma, and help neutralize free radicals through a series of redox reactions, which convert them into less reactive molecules. Superoxide dismutase (SOD) (EC 1.15.1.1), catalase (CAT) (EC 1.11.1.6), glutathione peroxidase (GPx) (EC 1.11.1.9) and glutathione reductase (GR) (EC 1.8.1.7) all play a key role in scavenging ROS [47], but their concentration is diminished during the dilution process before cryopreservation, which leaves spermatozoa vulnerable to oxidative stress. Additionally, they have been shown to function synergistically. SOD found in the cytoplasm and the mitochondria (Cu, Zn-SOD and Mn-SOD respectively) are effective at dismutating O_2_^•−^ to create H_2_O_2_ and O_2_. While still reactive, H_2_O_2_ is less damaging than O_2_^•−^ and can be subsequently broken down by other enzymatic mechanisms like those catalyzed by CAT or GPx into water [7]. While GPx requires the presence of reduced glutathione (GSH) to be active, CAT does not require any additional substrate to be available in order to function. Being a key substrate for GPx and GR, GSH is vital in neutralizing free radicals, with the result being the conversion of H_2_O_2_ into water [47,48]. Finally, GR helps regenerate GSH from its oxidized form (GSSG), which allows the cell to continue to use GPx as a neutralizing agent [49].

Many studies, mainly before 2019, have explored the addition of exogenous enzymatic antioxidants with mixed results. Del Prete et al. [49] and Das et al. [50] reported the positive effects of the addition of SOD, CAT, and GPx simultaneously on the viability and sperm motility of stallion and buck (goat) semen respectively; these studies from other species are cited as background only and were not part of the evidence set. The supplementation of SOD (25 and 100 U/mL) with GSH (2.5 and 7 mM) to ram semen extenders was tested by Silva et al. [51]^†^ and confirmed that, with proper dosage, the ultrastructures of both the acrosome and mitochondria were in better shape post-thaw, despite the difference in fertility after insemination not being significant. An interesting novel approach by Forouzanfar et al. [52]^†^ explored the use of manganese (III) meso-tetrakis (4-benzoic acid) porphyrin (MnTBAP), a synthetic enzyme that mimics SOD. While toxic at higher concentrations, MnTBAP improved ram sperm motility, acrosomal membrane integrity, and plasma membrane integrity post-thaw while also resulting in lower levels of cryocapacitation.

### 4.2. Nonenzymatic Antioxidants

Unlike enzymatic antioxidants, non-enzymatic antioxidants are usually exogenous, but this is not always the case. Sperm cells, including ram sperm, are highly specialized for motility and fertilization, so their energy and metabolic resources are usually geared towards supporting these functions rather than antioxidant defense systems. As a result, they have a limited capacity to synthesize and store non-enzymatic antioxidants. Some of the natural non-enzymatic antioxidants found in seminal plasma include vitamins A and C, GSH, carnitine, and pyruvate [53]. Levels of these antioxidants are lower than those found in other types of cells, and the addition of diluents to semen before freezing reduces their concentration even further. As such, adding exogenous antioxidants from external sources to extenders can play a critical role in mitigating the detrimental effects of ROS and help restore the redox balance of semen, which in turn could improve fertility outcomes [53,54]. Non-enzymatic antioxidants can be grouped based on various factors, such as water-soluble or lipid-soluble, but for the purpose of this review, they have been sorted into the following categories: vitamins, amino acids and their derivatives, plant extracts, and hormones and growth factors.

#### 4.2.1. Vitamins

Due to their antioxidant potential, recent studies have explored the addition of not just vitamins C and E, which are present in ram semen naturally, but also vitamin D, as a potential extender supplement (see Table 1). Aktar et al. [48] demonstrated that a supplement of 50 ng/mL of vitamin D3 improved sperm kinematic parameters, specifically total and progressive motility, as well as enhanced plasma membrane integrity and the membrane potential of mitochondria of frozen/thawed sperm. Total motility rose from 37.0 to 55.0% and progressive motility from 20.8 to 40.2%.

Vitamin E is a lipid-soluble vitamin that is very effective at removing ROS and breaking the chain reaction of lipid peroxidation by donating a hydrogen from a hydroxyl group. After neutralizing a free radical, vitamin E becomes oxidized, but it can be “recycled” back to its active form with the help of other antioxidants, including vitamin C, which can donate electrons to the oxidized vitamin E and restore it to its reduced, active state. This allows vitamin E to continue functioning as an antioxidant even when its concentration is low. Additionally, the vitamin radicals are less reactive than the scavenged ROS, which in turn does not perpetuate the radical chain reaction [58,60]. Diab et al. [46] found that vitamin E did not perform as well as aqueous rosemary and ginseng extracts as far as improving sperm recovery rates and reducing GSH levels, and cervical-mucus penetration was better with the plant extracts than with vitamin E or L-carnitine (each tested separately at 20 mg/mL). Espina-Ávila et al. [56] found that vitamin E improved the number of sperm with preserved membranes and improved both integrity and permeability. It should be noted, however, that Triladyl was used as the extender, while Diab et al. [46] used a Tris-based egg yolk extender.

Other studies have tested adding both vitamins E and C to both chilled and frozen extenders. According to Akhter et al. [55], vitamin C, vitamin E, or a combination of both improved a few motility parameters, but they had no effect on membrane integrity or viability when stored at 5 °C. In frozen–thawed semen, Rostami et al. [58] saw a significant improvement in sperm quality when vitamin E (2 mM, tested together with 100 mM trehalose; total motility rose from 46.0 to 62.4%) was supplemented, although no benefit was found when adding vitamin C. Trolox, a hydrophilic vitamin E analog, has also been shown to help preserve ram sperm motility during the cryopreservation (motility 35.0 to 50.0% with 1 mM), but more research on the use of this analog is needed [19].

A few recent studies have also explored using nanoemulsions of vitamin E as a way to overcome its low solubility in water without the use of ethanol or DMSO. In a comparative study on red deer, Sánchez-Rubio et al. [59] added vitamin E nanoemulsion at 6 and 12 mM to thawed stag sperm and incubated it for 4 h with or without induced oxidative stress. Without oxidative stress, 12 mM maintained total motility in line with that of the control (17.3 vs. 17.5%) but did not improve it. Under oxidative stress, it protected the acrosome (38.3 vs. 28.6% intact acrosomes) and reduced free radical production. It is important to note that this is a model of protection against oxidative stress, and it is not evidence with regard to rams. Jurado-Campos et al. [57] took a different approach with nanoemulsions by using 12 mmol/L vitamin E as a supplement to Andromed, a commercially available extender based on soybean lecithin, and incubating ram sperm without chilling or freezing at temperatures of 15 °C and 22 °C. In order to induce oxidative stress, they added 100 μmol/L Fe^2+^ and 500 μmol/L ascorbate. Without induced oxidative stress, the nanoemulsion was able to improve motility after 96 h at 15 °C (72.8 vs. 54.6%), but at 22 °C, the control had higher motility. Under induced oxidative stress, the nanoemulsion maintained viability and mitochondrial activity better than the control at both temperatures. Even more interesting was that after 96 h at 15 °C, sperm viability was comparable to the viability of the freshly ejaculated sperm. Both studies used 12 mM, but they differ in species as well as the choice of extender used.

#### 4.2.2. Amino Acids

Amino acids, besides being the building blocks of proteins, are considered to be good antioxidants. Functional groups can react and neutralize/stabilize free radicals by donating electrons or hydrogen atoms, thus breaking the chain reaction that occurs during oxidative stress. Amino acids with side chains that contain sulfur (thiol groups) or aromatic side chains have strong free radical scavenging abilities, with tryptophan and phenylalanine exhibiting the strongest ability to accept OH^•^ [61]. Supplementation of various amino acids, such as cysteine, cysteamine, methionine, and L-arginine, has been shown to reduce oxidative stress and improve sperm integrity, motility, and viability when supplemented pre-freeze and post-thaw, which suggests that enhancing antioxidant defenses can support the DNA repair processes in mature ram sperm [11,62,63,64].

Since 2019, many studies have explored the addition of amino acids and their derivatives to ram semen extenders (see Table 2), and the results have shown that the addition of GSH and cysteamine effectively enhances sperm quality during storage. GSH improved motility and antioxidant capacity across multiple concentrations during both chilled and frozen storage at concentrations from 0.5 mM up to values that were reported as 50–200 mM [sic] [65,66,67]. In frozen–thawed semen, 4–5 mM GSH increased total motility by about 8 to 13 percentage points (42.1 to 49.8% [65]; 48.7 to 61.6% after 3 h of post-thaw incubation [68]), but pregnancy did not differ from the control (20.0 vs. 30.0%) [65]. In another study, 5 mM had no effect [69], while using 5 mM in chilled semen increased motility from 70.6 to 87.2% after 48 h [67]. The use of arginine provided mixed results, which may be attributed to the choice of extender and/or concentration [62,70,71], while L-carnitine and L-glutamine exhibited no positive effects that could be attributed to their supplementation [68,72]. Kia & Vatankhah [73] found that D-aspartic acid at 10 mg/L provided the highest protective effect among the concentrations tested for membrane integrity and mitochondrial activity while increasing GPx and total antioxidant capacity and reducing MDA levels and sperm abnormalities. In frozen–thawed ram semen, 1 mM cysteine increased motility from 45.0 to 56.0% and acrosome integrity from 63.0 to 77.6% [11], and 1.0 mg/mL raised viability from 50.7 to 57.2% [63]. One promising and novel approach supplemented the extender with cysteamine loaded on selenium nanoparticles with surprising success, showing enhanced motility, viability, DNA and plasma membrane integrity, abnormality, and SOD activity, as well as reducing MDA levels [64].

#### 4.2.3. Quinones and Nitroxides

Quinones and nitroxides are two distinct classes of organic compounds that can interact or participate in chemical processes together. Both compounds play important roles in biological systems, as quinones are found in electron transport chains, while nitroxides can mimic biological free radicals. At low levels, quinones and nitroxides can act as signaling molecules, but higher concentrations can induce oxidative stress and lead to sperm damage and impaired functionality [74]. Thus far, the use of both classes of compounds together in ram sperm extenders has not been documented, but multiple studies have explored using various quinones or nitroxides as additives to semen diluents, as shown in Table 3.

In the context of quinones and semen extenders, ubiquinone (Coenzyme Q10; CoQ10), thymoquinone, pyrroloquinoline quinone (PQQ), and mitoquinone (MitoQ) have been tested for their antioxidant properties to enhance sperm preservation [75,76,77,81]. CoQ10, part of the mitochondrial respiratory chain, plays an important role in energy metabolism and functions as an antioxidant in cell membranes. One study has shown that supplementing CoQ10 to an extender with either soybean lecithin or egg yolk as a cryoprotectant preserved motility, membrane integrity and mitochondrial membrane potential compared to the control, regardless of the cryoprotectant used [76]. MitoQ, a CoQ10 derivative that has a lipophilic cation combined with the antioxidant component quinone, can pass through the cell and mitochondrial membranes, thanks to the lipophilicity provided by the cation. Like CoQ10, MitoQ showed higher mitochondrial membrane potential when added to semen extenders, but it also reduced lipid peroxidation and DNA damage, while preserving motility and viability and increasing the pregnancy rate after insemination (33.3 to 52.2%) [75]. Comparatively, thymoquinone, which is also lipophilic, exhibited similar effects but without any significant influence on oxidative stress levels [77].

In addition to CoQ10 and MitoQ, PQQ—which is water-soluble and has properties similar to combining ascorbic acid, riboflavin, and pyridoxal-5-phosphate into one molecule—is capable of acting as a free radical scavenger, as well as acting as a catalytic unit for dehydrogenases when oxidizing NADH [82]. When added to a boar sperm extender (comparative evidence) that had induced oxidative stress by low glucose conditions, PQQ enhanced ATP generation and improved linear motility [81]. It should be noted that PQQ has not been evaluated in ram semen in the studies included in this review.

Among nitroxides that also act as ROS scavengers, Mito-TEMPO (C_29_H_35_ClN_2_O_2_P) specializes in neutralizing ROS inside the mitochondria. One of the key features of Mito-TEMPO is its structure, which includes a nitroxide moiety that is conjugated to a triphenylphosphonium cation. This enables it to selectively accumulate in the mitochondria due to the negative membrane potential. Inside the mitochondria, it acts as an SOD mimetic, neutralizing superoxide radicals before they can increase to a level that impairs mitochondrial function and compromises sperm motility and viability [80,83]. This mitochondrial mechanism has been shown mainly in vitrified sheep oocytes and has not been measured directly in ram sperm [83]. Recent studies have all shown that doses between 5 and 50 µM of Mito-TEMPO had a positive effect on post-thaw motility, mitochondrial membrane potential, viability and acrosome and membrane integrity [78,79,80]. At 50 µM, total motility rose by 7.6–12.1 percentage points (37.0 to 44.6% [79]; 42.4 to 54.5% [80]), and pregnancy after insemination increased from 25.3 to 44.0% [80] and, with 40 µM, from 72.5 to 81.3% [78]. Zarei et al. [79] found that these same doses had no effect on DNA fragmentation or morphology, whereas Asadzadeh et al. [80] showed less DNA fragmentation, but with higher H_2_O_2_ levels. The study by Shi et al. [78] reported that the pregnancy rate after insemination with post-thaw sperm was higher, and more interestingly, they found that Mito-TEMPO upregulated 179 differentially expressed proteins, which are proteins that are mainly involved with motility, capacitation, and energy metabolism. Overall, the combined effects of nitroxides and quinones offer a multifaceted approach to managing oxidative stress, but consideration must be given to the nuanced interaction between nitroxides and quinones, which can be complex, as nitroxides can undergo oxidation to reactive oxoammonium compounds [84].

#### 4.2.4. Plant-Derived Antioxidants—Polyphenols

Plant-derived compounds as additives in semen extenders are rich in antioxidants and bioactive molecules, which offer a promising alternative to synthetic additives. The advantages of incorporating plant extracts include their potential to mitigate oxidative stress, reduce microbial contamination during storage, and improve sperm viability, motility, plasma membrane integrity and mitochondrial activity of ram spermatozoa [85,86], but many challenges still exist, including the variability in the effectiveness of different plant extracts, their potential cytotoxic effects at higher concentrations, and the need for standardized protocols to ensure consistent post-thaw results. Despite these obstacles, the integration of plant-derived compounds into semen extenders represents a natural and potentially effective strategy to improve the quality and longevity of stored ram semen [86,87].

Polyphenols are plant-derived compounds that are known for their strong antioxidant properties that act through several mechanisms, including the direct scavenging of free radicals, chelation of metal ions, inhibition of NADPH oxidases, and modulating cellular antioxidant responses by activating the nuclear factor erythroid 2-related factor 2/antioxidant response element (NRF2/ARE) pathway in somatic cell models [86]. Despite their potent antioxidant properties, polyphenols are also able to exhibit pro-oxidant activities under certain conditions, such as high concentrations or specific pH levels, which can lead to the creation of ROS and oxidation of transition metals. This dual role highlights the complexity of polyphenol reactions, which must be considered when using them as supplements to semen extenders [86,87].

Polyphenols can be broken down into two main groups: flavonoids and non-flavonoids. Flavonoids are characterized by a 15-carbon skeleton consisting of two phenyl rings that are connected by a heterocyclic ring to form a C6-C3-C6 structure [86]. Recent studies have explored the addition of quercetin, myricetin, rutin, baicalein, and hesperidin to ram semen extenders for cryopreservation using a Tris–egg yolk extender (see Table 4). One study found that the addition of quercetin at a concentration of either 10 or 20 μM improved motility parameters, while supplementing 10 μM of quercetin with 1 mM of extracellular ATP significantly improved the pregnancy rate after laparoscopic insemination (58.1 vs. 27.5%) [88]. Conversely, another study with multiple concentrations of myricetin exhibited no benefits to motility and no antioxidant effects [89]. This was similar to baicalein, which did not improve the total antioxidant capacity, but it did preserve motility and reduce chromatin damage compared to the control, when supplemented at 0.5 mM. Higher doses were found to produce negative effects [90]. Najafi et al. [91] found that 0.75 and 1 mM rutin not only significantly improved motility, mitochondrial activity and membrane integrity, but also increased ATP levels and antioxidant capacity, while reducing ROS levels and apoptosis. Supplementation with hesperidin, a citrus-derived flavonoid, demonstrated protective effects on spermatozoa at 10 µg/mL by maintaining acrosomal integrity and decreasing lipid peroxidation, and at both 10 and 50 µg/mL concentrations, it prevented chromatin damage [92].

Non-flavonoid polyphenols encompass a variety of structures distinct from the flavonoid backbone, including phenolic acids, such as caffeic and gallic acids; stilbenes like resveratrol; and lignans found in seeds and grains. These compounds vary in their carbon frameworks and functional groups, which contribute to their unique biological activities [86]. Table 5 shows an overview of recent studies involving non-flavonoid polyphenols and semen extenders. Resveratrol, found in grapes and berries, provides mitochondrial protection and reduces ROS production, but the results when added to ram semen extenders have varied.

During chilled storage with Triladyl as the extender, no effects whatsoever were seen with regard to sperm parameters in the first 5 days, but after 15 days of storage both groups with 200 or 400 μM of resveratrol showed better motility and higher survival rates [93]. Brair et al. [13] found that 50 μM/mL [sic] of resveratrol increased sperm binding to the perivitelline membrane of hen eggs, an in vitro indicator of fertilizing potential, while Zhu et al. [10] saw that the same concentration significantly enhanced acrosome and membrane integrity, antioxidant capacity, mitochondrial membrane potential, and reduced DNA damage. Trans-ferulic acid and chlorogenic acid have both been used for chilled storage, increasing antioxidant capacities at the most favorable concentration among those tested, with trans-ferulic acid showing negative effects once that dose was surpassed. It should be noted that while trans-ferulic acid is not classified as a polyphenol according to standard definitions, its methoxy group has similar antioxidant properties. As such, it has been included in this section [94,95]. Gallic acid, widely found in walnuts, hazelnuts, grapes, berries, carrots and more, resulted in greater total motility post-thaw when added at a concentration of 2 mM when compared to the control group and other concentrations, without any additional benefit [96].

**Table 5:** Non-flavonoid polyphenols in semen extenders.

Reference	Species and Sperm Source	Model	Supplement(s)	Concentration in Extender (as Reported)	Extender	Main Outcomes	Endpoint
Salimi et al. [95]	Ram, ejaculated	Chilled	t-FA ^a^	2.5, 5, 10, and 25 mM	Tris–egg yolk	In total, 10 mM t-FA gave higher antioxidant levels and motility at 72 h. In total, 25 mM t-FA increased malondialdehyde and showed the lowest motility/viability.	E2
Wang et al. [94]	Ram, ejaculated	Chilled	Chlorogenic acid	0.2, 0.4, 0.8, and 1.2 mg/mL	Tris–egg yolk	Among the concentrations tested, 0.8 mg/mL was the most favorable, with the highest acrosomal/membrane integrity, the highest levels of catalase, superoxide dismutase, adenosine triphosphate and total antioxidant capacity.	E2
Al-Mutary et al. [93]	Ram, ejaculated	Chilled	Resveratrol	200 or 400 μM	Triladyl	No effect on sperm parameters in storage times 0, 24, 72 or 120 h from either concentration, but total and progressive motility and survival time were higher for both concentrations of resveratrol than the control at day 15.	E1
Brair et al. [13]	Ram, ejaculated	Cryopreserved	Resveratrol	10 and 50 µM/mL [sic]	Tris–egg yolk	No effect on motility, viability, or membrane/acrosome integrity, but 50 μM/mL [sic] of resveratrol increased sperm binding to the perivitelline membrane of hen eggs (in vitro).	E3
Zhu et al. [10]	Ram, ejaculated	Cryopreserved	Resveratrol	25, 50, 75, and 100 μM	Tris–egg yolk	In total, 50 μM was the most favorable among those tested, increasing total motility (37.6 to 69.2%) and progressive motility (23.6 to 48.4%), while enhancing acrosome and membrane integrity, antioxidant capacity and mitochondrial membrane potential. In total, 100 μM had negative effects (progressive motility 19.1%, below the control).	E2
Gungor et al. [96]	Ram, ejaculated	Cryopreserved	Gallic acid	0.05 or 2 mM	Tris–egg yolk	In total, 2 mM gallic acid resulted in greater total motility post-thaw than the control group. No other parameters were affected.	E1
Arando et al. [97]	Ram, ejaculated	Cryopreserved	Olive oil-derived ^b^	10, 30, 50 and 70 μg/mL (separately and mixed)	Biladyl	HT + DHPG mixture negatively affected membrane/acrosome integrity. HT, DHPG or both significantly reduced lipid peroxidation with similar values to fresh sperm, without positively affecting any other values.	E2

^a^ Trans-ferulic acid; ^b^ hydroxytyrosol (HT) and 3,4-dihydroxyphenylglycol (DHPG). E1, sperm-quality variables (motility, kinematics, viability, membrane, acrosome and mitochondrial integrity, DNA integrity); E2, oxidative and biochemical markers (reactive oxygen species, malondialdehyde, antioxidant capacity and enzymes); E3, in vitro functional tests (cervical-mucus penetration, oocyte binding, in vitro fertilization); E4, in vivo fertility (pregnancy or lambing rate). [sic] denotes a unit reproduced exactly as printed in the original article after verification against it; such values may contain errors in the original and should be interpreted with caution.

A study in Spain tested the addition of two olive oil-derived antioxidants, hydroxytyrosol (3,4-dihydroxyphenylethanol, HT) and 3,4-dihydroxyphenylglycol (DHPG), which were extracted from olive pulp, a waste product from the production of olive oil. The results of this study showed that supplementing either HT or DHPG to Biladyl, a commercial extender, significantly reduced lipid peroxidation levels post-thaw to values similar to fresh sperm, but when added together, the supplement negatively affected both membrane and acrosome integrity [97].

Additionally, many plant-derived antioxidants contain both flavonoid and non-flavonoid polyphenols, which can offer a synergistic approach to combating oxidative stress and provide unique benefits due to their diverse phytochemical profiles (see Table 6). *Basella rubra* fruit extract (fresh fruit juice), which is rich in betacyanins, bioflavonoids, carotenoids, and β-sitosterol, improved sperm cryopreservation at a concentration of 0.6% when compared to the control group and other tested concentrations, but 1.6% did not differ from the control. When pre-freeze values were taken into account, only 0.8% remained above the control. The loss of effect at higher concentrations might be due to the acidic nature of the fruit and the high content of antioxidants [98]. Khalil et al. [99] recently tested various concentrations of quinoa seed extract (aqueous extract; total phenolic, flavonoid, and tannin contents were quantified) with a Tris–soybean lecithin extender and found that all concentrations were able to reduce MDA levels and improve total antioxidant capacities by promoting antioxidant-related genes, but concentrations of 750 and 1000 µg/mL gave the most favorable results among those tested for viability and motility, while concentrations between 500 and 1000 µg/mL of the seed extract reduced apoptotic-like changes.

Ginger (*Zingiber officinale*), echinacea (*Echinacea purpurea*) and fennel (*Foeniculum vulgare*) extracts have also been tested as extender supplements. Ginger and echinacea have been considered medicinal plants, as both have been utilized in medicine and nutrition due to their high numbers of antioxidant compounds. Ginger is rich in gingerols, shogaols (which provide anti-inflammatory and antimicrobial properties), catechins and flavonols that contribute to its antioxidant capacity, while echinacea is full of immune-supporting flavonoid glycosides and compounds known for their immunomodulatory effects, like alkamides and caffeic acid derivatives [100,101]. A study by Merati & Farshad [100] found that supplementing extenders with aqueous extracts of either ginger or echinacea, 10 mg/L and 20 mg/L respectively, was able to not only preserve sperm viability and both membrane and acrosome integrity, but they were able to also significantly lower MDA levels compared to the control group. Najafi et al. [101] tried adding different concentrations of aqueous fennel extract (5, 10 and 15 mg/L) to a Tris–soybean lecithin extender with varying concentrations of spermatozoa (200 and 400 × 10^6^ sperm/mL). They obtained the highest quality of post-thaw sperm among the combinations tested by using 10 mg/L of fennel extract with 200 × 10^6^ sperm/mL (total motility 51.3 to 60.4%), the more favorable of the two sperm concentrations tested. This suggests that the additive’s effect can therefore depend on sperm concentration.

Another promising source of antioxidants comes from the plant *Moringa oleifera*, also known as the drumstick tree, as shown in Table 7. The drumstick tree is originally native to India and is used extensively across Southeast Asia and Africa as a vegetable, as herbal medicine and even for water purification. Extracts from its seeds and leaves differ significantly in their chemical compositions. Leaf extracts are rich in polyphenols, flavonoids, vitamins and minerals, with some of the key active compounds being quercetin, kaempferol, and chlorogenic acid [102]. Seed extracts, on the other hand, are notable for their high oil content, which is made up of about 39% fat (a significant portion being monounsaturated fatty acids like oleic acid), and levels of proteins, sterols, and tocopherols [103].

El-Seadawy et al. [18] and Shokry et al. [17] both used *Moringa oleifera* leaf extracts as supplements at similar concentrations in a Tris–egg yolk extender and found that about 0.6 mg/mL was the most favorable concentration among those tested (total motility 40.0 to 55.2% with 0.64 mg/mL [18]; motility 42.0 to 62.0% with 600 mg/mL [sic] [17]), whereas El-Seadawy et al. [18] found 0.48 mg/mL to be harmful. Additionally, Shokry et al. [17] found that this concentration increased the pregnancy rate after insemination from 28.6% to 52.4%. It should be noted, however, that both studies used different solvents during the extraction process, methanol and ethanol respectively. Methanolic extracts generally exhibit higher total phenolic content and ferric reducing antioxidant power, while ethanol extracts have higher concentrations of bioactive compounds such as alkaloids, flavonoids, tannins, and phenols [105]. The extracts are therefore not equivalent, and their concentrations cannot be compared directly. As such, in vivo testing with the methanolic leaf extract would allow for a more accurate comparison of the two studies. Unlike the use of *Moringa oleifera* leaf extract, the use of the seed extract has been even less documented. One study showed that antioxidant capacity post-thaw was increased with doses of 0.5 and 5.0 mg/mL Moringa seed extract (80% methanol extract of defatted seeds), but only 0.5 mg/mL was able to preserve viability and motility, while decreasing membrane damage [104].

#### 4.2.5. Hormones and Growth Factors

The incorporation of hormones into semen extenders for cryopreservation has recently shown promise, not only in enhancing sperm viability and function post-thaw but also in improving motility and reducing apoptotic-like changes [20,62,106]. However, despite the organic farming perspective, which has gained popularity in recent years, synthetic hormones are strictly prohibited for growth or reproduction [107]. Traditionally, hormones are defined as chemical messengers that are produced by specialized cells and play a crucial role in regulating physiological processes, but this definition has evolved to encompass a broader range of signaling molecules including cytokines and growth factors, which can also function as hormones under certain conditions [108]. By binding to specific receptors on target cells, hormones are able to initiate cellular responses that help maintain homeostasis and coordinate complex functions or processes; as such, hormones could possibly help mitigate cryodamage through various mechanisms (see Table 8) [109].

Melatonin (N-acetyl-5-methoxytryptamine) is a powerful antioxidant that is synthesized and secreted in the pineal gland of mammals [109] and can stimulate mitochondrial respiration and ATP synthesis by increasing the activity of electron transport chain complexes I and IV, which in turn improves its efficiency and reduces ROS levels (evidence from human spermatozoa and other models) [111,115]^†^. In human sperm (comparative evidence), it was reported that a 3 mM supplement of melatonin to the semen extender significantly improved motility and viability and reduced ROS levels, caspase-3 (EC 3.4.22.56) (a key mediator of apoptosis) activity and the total number of dead sperm cells while increasing protein kinase B (AKT) (EC 2.7.11.11) phosphorylation compared to the control group. This suggests that melatonin may act via the phosphoinositide 3-kinase (PI3K)/AKT signaling pathway, which regulates cell survival, growth and metabolism and can inhibit pro-apoptotic proteins, thereby preventing programmed cell death [115]^†^. In ram sperm, Fang et al. [20] showed that melatonin activates melatonin receptor 1 (MT1), which, in turn, stimulates the PI3K/AKT signaling pathway, leading to the inhibition of glycogen synthase kinase-3 beta (EC 2.7.11.26). This resulted in the suppression of mitochondrial permeability transition pore (mPTP) openings, helping preserve mitochondrial integrity and enhancing sperm quality and pregnancy rate.

In addition to melatonin’s ability to act through cell signaling pathways, melatonin is also a potent free radical scavenger due to the pyrrole ring in its structure and can inhibit the enzyme nitric oxide synthase (EC 1.14.13.39), which prevents lipid peroxidation reactions in general [116]. Multiple studies have tested the addition of various concentrations of melatonin to different semen extenders with contrasting results. Li et al. [111] found that enzymatic antioxidant activity was increased, and motility and membrane integrity were preserved with 0.5 mM, the most favorable concentration among those tested, while Shahat et al. [62] had better results with 1 mM of melatonin. The two studies used Optidyl and Steridyl, both commercial extenders, respectively. One study found that even a small dose of 0.3 mM helped improve the preservation of motility and viability when added to a Tris–egg yolk extender [110]. Carrico et al. [69] found that 1 mM improved only progressive motility (20.5 to 26.7%), with no effect on total motility or viability. Gains in motility increased from 44.0 to 66.1% with 0.5 mM [111] and from 13.9 to 51.1% with 0.1 µM [20]. Fang et al. [20] also reported a higher pregnancy rate (34.8 vs. 16.0%).

Recent novel approaches using insulin-like growth factor I (IGF-I), insulin and erythropoietin have been tested for other species, including bulls and boars, but they have not yet been well documented with ram semen extenders [113,114]. IGF-I can be naturally found in semen and has been shown to play a role in the regulation of protein phosphorylation and acceleration of sperm cell energy metabolism. Furthermore, studies have found it to have a direct effect on SOD [114]. Pinto et al. [113] found that neither 100 nor 200 ng/mL of IGF-I showed any benefit with bull sperm. Oliveira Resende et al. [114] showed increased antioxidant potential as well as post-thaw sperm quality with only 30 ng/mL in boar sperm, suggesting either dose- or species-dependent benefits. Additionally, the addition of 150 µUI/mL [sic] of insulin improved preservation of motility, plasma membrane integrity, and mitochondrial membrane potential when added to multiple extenders [113]. Erythropoietin, a peptide hormone secreted by the kidneys that stimulates erythropoiesis, has also been found in reproductive tissues of both sexes and shown to exhibit antioxidant-like effects. While the exact mechanisms of its antioxidant action are still not clarified, the addition of erythropoietin to bull semen extenders showed a moderate effect on O_2_^•−^ levels and motility after thawing, but with higher doses, it did act as a capacitation factor [112]. All three are comparative evidence, and more research needs to be done to determine if these novel approaches could provide the same benefit to ram spermatozoa during cryopreservation.

## 5. Fatty Acids and Lipids

Fatty acids (FAs) play a crucial role in maintaining the structural and functional integrity of sperm cells during the cryopreservation process. Studies indicate that adding PUFAs may benefit sperm cells by stabilizing membranes under cryogenic conditions, though balancing antioxidant levels is essential to counteract peroxidation risks [117].

Ofosu et al. [117] found that specific PUFAs, namely arachidonic acid (AA), linoleic acid (LA), and docosahexaenoic acid (DHA), are vital for sperm survival during cryopreservation. Furthermore, research indicates a strong correlation between the fatty acid composition of spermatozoa and their motility and viability after thawing. Specifically, higher levels of n-3 PUFAs, particularly DHA, are directly correlated with improved sperm parameters post-cryopreservation. Conversely, monounsaturated FAs showed an inverse correlation [118].

The mechanisms by which FAs exert their protective effects on cryopreserved ram semen involve several interrelated processes. Besides acting as antioxidants and scavenging ROS, FAs help maintain the structural integrity of the sperm cells and preserve their functionality [117]. Secondly, the incorporation of FAs into the sperm plasma membrane can alter its fluidity and permeability, making it more resistant to the formation of ice crystals and the resulting osmotic stress. This helps prevent the disruption of the membrane and the leakage of essential cellular components during the freezing and thawing process [118]. Furthermore, in ruminants, certain FAs, such as DHA and eicosapentaenoic acid (EPA), have been proposed to influence the expression of genes involved in apoptosis, oxidative stress, and energy metabolism, which may help maintain the viability and functional capacity of the sperm cells post-thaw [119]; however, such effects were not assessed in the ram extender studies in this review.

Recent studies have explored the use of various FAs and lipids as additives to ram semen extenders, with varying degrees of success (see Table 9). Oleic acid, when combined with trehalose, significantly improved total motility and viability of ram sperm, while reducing lipid peroxidation and increasing SOD levels, indicating better oxidative stress management [120]. In a 2013 ram study, Towhidi et al. [14]^†^ also confirmed that n-3 FAs (1 ng/mL) combined with α-tocopherol (0.1 mM) enhanced sperm quality during freezing by increasing post-thaw progressive motility from 29.0 to 32.5% without changing total motility and raising the DHA content of the sperm membrane before freezing. Similarly, lipid mixtures from liposome kits, alone and when used with melatonin, demonstrated protective effects on sperm motility and DNA integrity in Angora goat sperm (comparative evidence) [121].

Novel strategies utilizing β-cyclodextrin (β-CD) as a carrier compound to enhance the solubility of lipids and fatty acids have shown promise in enhancing the cryopreservation of ram semen. β-CD is a cyclic oligosaccharide composed of seven glucose units linked by α-1,4-glycosidic bonds. It is known for its ability to form inclusion complexes with various hydrophobic compounds, enhancing their solubility and stability in aqueous environments [125]. Mortazavi et al. [122] found that the concentration and use of β-CD when adding oleic acid to Tris–citric acid–egg yolk extender gave the highest resistance to thermal shock and freezing at 0.25 and 0.50 mM. Furthermore, cholesterol, particularly when delivered via cholesterol-loaded cyclodextrin (CLC), has been shown to enhance the quality of ram semen extenders used for cryopreservation. Inanc et al. [123] demonstrated that CLC, when combined with trehalose, improved post-thaw motility, plasma membrane integrity and mitochondrial membrane potential. A dose of 2.5 mg per 120 million sperm provided the most favorable results among the concentrations tested.

Additionally, the most favorable concentration of fatty acid/lipid supplements appears to be species-specific and may vary depending on the composition of the semen extender and the cryopreservation protocol used [119].

## 6. Sugars

Sugars are a natural component of seminal plasma, with the most prevalent saccharide being fructose. Due to their unique chemical structure, different sugars play varying roles and have different impacts when used in semen extenders, as shown in Table 10. Some of the most common sugars used are fructose, glucose, sucrose, and trehalose, but raffinose and sugar alcohols, for example, sorbitol and mannitol, have also been tested [126,127]^†^. Sugars not only provide an energy source for spermatozoa but also help maintain osmotic balance and membrane support throughout the freeze–thaw process [126]^†^.

Glucose and fructose are two of the most common monosaccharides that are used in semen extenders. Both are metabolized by sperm cells via distinct pathways that can influence sperm function and energy production. Studies on boars and other species have found that fructose often plays a larger role in metabolic processes in comparison to glucose, which can be attributed to differences in hexokinase (EC 2.7.1.1) activity and the transport mechanisms that are involved in the uptake of these sugars. Interestingly, glucose and fructose are utilized nearly the same when added separately, but ram sperm prefers to use glucose when both monosaccharides are available. This has been well documented and is thought to be because of the greater affinity of glucose for hexokinase [134,135]^†^.

Both fructose and glucose play a major role in sperm cell metabolism, as they are used to produce ATP. Matsuoka et al. [134]^†^ proposed that information about the changing relationship between fructose and both glucose and testosterone found in blood could be useful for determining proper doses of saccharide supplementation in semen extenders, since testosterone stimulates reproductive accessory glands to synthesize fructose from blood glucose, and rams exhibit seasonal changes in both scrotal size and testosterone levels, which de facto influence fructose concentration in seminal plasma. Ahmed et al. [21] demonstrated in epididymal ram sperm that the use of fructose at a concentration of 1.25% (*w*/*v*) outperformed glucose of the same concentration.

Trehalose and sucrose are the most common disaccharides used in semen extenders, each affecting sperm motility and viability differently. Unlike glucose and fructose, trehalose and sucrose are unable to cross the plasma membrane, and as such provide extracellular protection during cryopreservation. During freezing, both exhibit a unique “glass-forming” ability, which means they are not sharp when frozen, so they do not mechanically damage sperm cells unlike ice crystals [130,133]^†^. Furthermore, the unique structures of trehalose and sucrose help regulate the fluidity and stability of the plasma membrane by forming hydrogen bonds between the hydroxyl groups of the sugar and the phospholipids’ polar heads, which allows the sugar to replace water as dehydration occurs during freezing [127].

Studies involving sucrose and raffinose as additives in ram semen extenders are not well documented, but Herdis et al. [130] showed that 0.2 g/100 mL sucrose improved viability (52.7 to 62.8%) and membrane integrity. Motility improved only at 0.4–0.6 g/100 mL. In goat sperm (comparative evidence), Nurlatifah et al. [127] found that 0.6% sucrose more effectively maintained the quality of frozen sperm when combined with a Tris-based egg yolk extender than dextrose or raffinose.

Contrarily, trehalose as a ram semen extender supplement has been extensively explored. Özmen et al. [129] reported a positive influence of trehalose on motility and membrane integrity, similar to an earlier study from 2018 by Gungor et al. [133]^†^. Özmen et al. [129] observed that the addition of 25 and 50 mmol/L trehalose significantly protected spermatozoa motility for up to an hour post-thaw but not 2 or 4 h. They obtained the most favorable results (a protective effect on both progressive motility and integrity throughout the entire thaw period of 1, 2, and 4 h) with a combination of 25 mmol/L trehalose and 5% iodixanol, which was originally designed as an X-ray contrast but is now used to provide a gradient of densities due to its nonionic and inert structure. Contrastingly, in ram semen stored at 0 °C rather than frozen, Zhao et al. [128] found that 5–20 mM trehalose maintained acrosome integrity better than the control (73.9 vs. 51.3% after 12 days with 5 mM), with no difference between concentrations and no improvement in progressive motility. Furthermore, pregnancy after insemination using semen stored for 9 days was unchanged (66.0 vs. 65.7%). The results of these studies cannot be reconciled on dose alone, as they differ in extender, trehalose concentration, storage type and endpoints. Further research is needed in order to be more conclusive on its exact positive effects.

Recent novel approaches have explored the use of tree gums as an alternative all-natural source to synthetic saccharides, as their abundant availability, sustainability, biosafety, nontoxicity, and capability to be easily chemically altered have been intensively studied [132]. The structure of gums varies depending on the source, but it is generally composed of a heterogeneous mixture of saccharides joined by glycosidic bonds, with protein and phenolic components sometimes interspersed throughout. They are soluble in water and can form gels, thanks to the inclusion of uronic acids in the form of pyranose, which allows them to store water [136]. Shehab-El-Deen et al. [131] hypothesized that gum arabic (*Acacia senegal*), comprising galactose, rhamnose, glucuronic acid and arabinose, could be used as a replacement for egg yolk in a Tris-based extender. The results showed comparable sperm motility, membrane integrity and viability to results of the same extender used with egg yolk and suggest that gum arabic could be used as a substitute.

Apricot tree gum (*Cytospora cincta* sacc), another promising natural additive with dual functionality, is composed of galactose, xylose, mannose, rhamnose and phenols of hydroquinone, catechol and pyrogallol [132]. Its sugars provide an energy source during cryopreservation and help prevent the formation of intracellular ice crystals, while the phenolic components exhibit antioxidant properties and inhibit the formation of ROS. Unlike Shehab-El-Deen et al. [131], Khanzadeh et al. [132] did not try to replace egg yolk in a Tris-based extender but rather supplement it with 10, 15 and 20 mg/mL apricot tree gum. There were no significant differences post-thaw in sperm cell motility, abnormality and viability between the various apricot tree gum concentrations, but each concentration performed better than the control group. Furthermore, the extenders with apricot tree gum had lower levels of MDA than the control group, which suggests their antioxidant activity.

## 7. Conclusions

This review aimed to determine the current state of the art of cryopreservation techniques for ram semen with a special emphasis on the role of semen extender additives on post-thaw sperm quality. The results revealed that the major issues that arise due to cryopreservation are oxidative stress, ice crystal formation and structural damage to the plasma and acrosomal membranes. Despite improvements in semen extenders, the fertility rates of artificial insemination using frozen–thawed ram semen are still low, which indicates that conventional extenders are not able to provide sufficient protection against cryoinjury.

The effectiveness of cryoprotective agents is based on two kinds of protection. Penetrating and non-penetrating cryoprotectants act mainly physically, in that they limit intracellular ice formation and osmotic stress by stabilizing proteins and membranes (for example, through “preferential exclusion theory”), but most of the additives reviewed here act biologically by limiting oxidative damage, modulating cell signaling or supporting mitochondrial function. Many of these biological mechanisms were described in other species or cell models and should be regarded as hypotheses to be tested in ram spermatozoa rather than established mechanisms. Current research trends are shifting toward the use of both synthetic and natural additives to enhance the effectiveness of cryopreservation methods. For instance, natural antioxidants like polyphenols, plant extracts, and vitamins have been shown to have the ability to reduce oxidative stress and thus maintain sperm motility and viability. However, these compounds exhibit bimodal activity. At elevated concentrations or specific pH levels, their pro-oxidant effects can induce additional ROS production and even chromatin damage. All-natural tree gums provide multifunctional benefits as both antioxidants and metabolic substrates. The most consistent evidence in rams is for GSH, vitamin E, cysteine, Mito-TEMPO and melatonin in frozen–thawed semen. Most other additives rest on single studies with sperm-quality endpoints, and only a few studies measured in vivo fertility. Sperm-quality and oxidative-stress endpoints are surrogate measures. As such, an improvement in them does not establish an improvement in fertility.

Due to possible contamination risks from the use of egg yolk, studies are looking for alternative cryoprotectants like soybean lecithin or *Gossypium* spp. and *Sesamum indicum* oils (both oils tested only in chilled storage), all of which were used to replace egg yolk in extenders with comparable viability and motility results in their respective storage models. Nanoparticles and nanoemulsions have been successfully used to improve the delivery of poorly water-soluble antioxidants (vitamin E nanoemulsions; cysteamine-loaded selenium nanoparticles), and there is also a growing number of studies creating synthetic alternatives to naturally occurring compounds (Trolox, MitoQ, and Mito-TEMPO), which may provide more precisely defined concentrations and more compositional consistency than natural extracts.

Contradictory results and the lack of transferable doses share a similar cause. Many of the studies differed in several respects: storage model (chilled vs. frozen), base extender (egg yolk and soybean lecithin bind additives differently), sperm concentration, extraction method for plant products, endpoints, and times assessed after thawing. For example, a vitamin E nanoemulsion improved ram sperm motility after 96 h of storage at 15 °C, while at 22 °C the control retained higher motility after 48 h, and melatonin was most favorable at 0.5 mM in Optidyl but at 1 mM in Steridyl. Concentrations were also reported in incompatible units, and most studies used few males. Therefore, a concentration that was most favorable in one study should not be read as a transferable dose.

Despite these advancements, several research gaps still remain. More research is needed to optimize the concentrations of additives, especially those derived from natural sources (particularly concerning the variability from solvent extraction, i.e., methanolic vs. ethanolic), and to find the most effective combinations. Furthermore, future studies should report concentration-response curves instead of just single “best” concentrations. Since additives are tested as concentrations in vitro, the appropriate parameters are the half-maximal effective concentration (EC50), which is defined as the concentration that produces 50% of the maximum improvement compared to the control in a predefined endpoint of sperm function (e.g., post-thaw progressive motility), and the half-maximal toxic concentration (TC50). TC50 is defined as the concentration at which a predefined measure of cell integrity (for example, post-thaw plasma membrane or DNA integrity) falls to 50% of the control value. A decline in such endpoints may indicate, but does not prove, cytotoxicity. Furthermore, we propose the ratio TC50/EC50 as a framework for future standardized concentration-response studies rather than a validated metric. It would describe the window between benefit and toxicity. In order for the metric to be comparable across additives and experimental designs, we suggest that a minimum of five concentrations (on a logarithmic scale) of each additive be tested, in the same base extender, sperm concentration, and storage model. The male should be selected as the statistical unit, and concentrations should be expressed in molar units (for plant extracts, standardization is a must). If the additive has biphasic effects, it might not fit a standard sigmoid curve, and in this case, we suggest that the range of concentrations that improves on the specified endpoint without reducing the predefined cell marker be reported instead.

Additionally, there is limited information on the effectiveness and stability of the new formulations after long-term storage. Studies should address long-term viability and kinematic parameters of new formulations after long-term storage, greater than 12 months, in liquid nitrogen. Many studies are limited to testing the new formulations on one or two breeds, which are locally available; as such, cross-breed validation is lacking. Most studies use locally available breeds with physiological variations (membrane lipid composition, metabolic rates, etc.). These variations are vital in determining the most suitable formulation for a specific breed. Breeds whose sperm membranes contain more PUFAs may differ in post-thaw viability, while breed hormonal variations (seasonal vs. non-seasonal breeders) can influence the doses of additives needed to maintain osmotic balance. Furthermore, the mechanisms underlying sperm recovery post-thaw are still not fully understood. To this end, it is important that these knowledge gaps be filled in order to improve the effectiveness, reliability, and scalability of cryopreservation protocols for ram semen.

## 8. Future Directions

Innovative semen extender formulations and optimized freezing and thawing protocols are the keys to successful artificial insemination programs and genetic conservation efforts. Research should support the sustainable management of livestock and genetic resources by reducing reliance on traditional egg-yolk-based extenders and embracing novel strategies. While none of the studies mentioned in this review can be directly compared to each other, due to the variations in extender compositions and interspecies variations amongst the breeds used, they do provide insights about what could work and what might not, providing direction for future studies. While the most suitable semen extender formulation for one breed might not be ideal for another, it can provide a baseline to which breed-specific adjustments can be applied. Combining many of the additives reviewed here into a single extender would not be expected to compound their benefits for many reasons. Several compounds are beneficial only within a narrow concentration range and become pro-oxidant or toxic above it (polyphenols, trans-ferulic acid, and resveratrol). Furthermore, combinations can interact. HT and DHPG were beneficial alone but harmed membrane and acrosomal integrity when combined; nitroxides can be oxidized to reactive oxoammonium compounds in the presence of quinones. Future studies should therefore test combinations alongside every single additive to distinguish synergistic and antagonistic effects.

In order to advance reproductive biotechnology in sheep, a shift from descriptive additive studies to standardized and mechanism-focused protocols is required. As such, the findings and recommendations presented in this review provide a foundation for new studies to help advance reproductive biotechnology in not just sheep but also other livestock species.

## Figures and Tables

**Table 1:** Vitamins in semen extenders.

Reference	Species and Sperm Source	Model	Supplement(s)	Concentration in Extender	Extender	Main Outcomes	Endpoint
Akhter et al. [55]	Ram, ejaculated	Chilled	Vitamins E, C	1 mg/mL	Tris, fructose, glucose, egg yolk	Supplementation of vitamin C, vitamin E, or a combination of both at 1 mg/mL had no beneficial effect during cold storage on viability or membrane integrity, but they improved some motility parameters.	E1
Diab et al. [46]	Ram, ejaculated	Chilled and cryopreserved	Vitamin E	20 mg/mL [sic] (table in paper shows 20 mg/100 mL)	Tris, sodium citrate, citric acid, fructose, egg yolk, (also tested rosemary and ginseng aqueous extracts and L-carnitine)	Ram sperm characteristics, sperm recovery rate, and reduced glutathione (GSH) levels were significantly higher in extenders supplemented with natural antioxidants (rosemary and ginseng) compared to control, and penetration ability of ram sperm into ewe cervical mucus was significantly better with vitamin E and L-carnitine	E3
Espina-Ávila et al. [56]	Ram, ejaculated	Chilled and cryopreserved	Vitamin E (α-tocopherol)	10 mg/mL [sic]	Triladyl	Chilled: Motility unchanged; membrane integrity and hypo-osmotic swelling (HOST) response higher at 0 and 24 h. Cryopreserved: Integrity and HOST response higher at 0 h with a small difference after thawing	E1
Jurado-Campos et al. [57]	Ram, ejaculated	Transport/Ambient and oxidative-stress challenge	Vitamin E nanoemulsion	12 mmol/L (12 mM)	Andromed	Without induced oxidative stress, 12 mM improved motility after 96 h at 15 °C (72.8 vs. 54.6%), while at 22 °C the control had higher motility after 48 h; under oxidative stress, 12 mM maintained viability and mitochondrial activity better than the control	E2
Aktar et al. [48]	Ram, ejaculated	Cryopreserved	Vitamin D3	12.5, 25, 50, and 100 ng/mL (dissolved in ethanol)	Tris, citric acid, fructose, trehalose, egg yolk	The 50 ng/mL vitamin D3 group exhibited significantly higher total and progressive motility, as well as improved sperm kinematic parameters compared to other groups, and enhanced plasma membrane integrity and mitochondrial membrane potential	E1
Rostami et al. [58]	Ram, ejaculated	Cryopreserved	Vitamins E, C	1 and 2 mM	Tris, fructose, glycerol, egg yolk, a sugar (trehalose, sucrose, or raffinose), taurine	Vitamin E (2 mM, tested together with a sugar) improved sperm quality (with trehalose, total motility 46.0 to 62.4%). Vitamin C and taurine gave no benefit.	E1
Yeni et al. [19]	Ram, ejaculated	Cryopreserved	Trolox (vitamin E analog)	1 mM	Tris-based, egg yolk	Post-thawed motility ratio was higher in 1 mM Trolox and 50 mM taurine groups compared with the control, and taurine had the highest plasma membrane integrity.	E2
Sánchez-Rubio et al. [59] ^‡^	Stag, epididymal	Oxidative-stress challenge	Vitamin E nanoemulsions	6 and 12 mM	Bovine Gamete Medium	Without oxidant, 12 mM Vitamin E nanoemulsion maintained motility at control level (17.3 vs. 17.5% at 4 h), while 6 mM and Trolox reduced it. Under induced oxidative stress, 12 mM protected acrosomes (38.3 vs. 28.6%) and reduced free radical production.	E2

^‡^ Denotes a comparative study. E1, sperm-quality variables (motility, kinematics, viability, membrane, acrosome and mitochondrial integrity, DNA integrity); E2, oxidative and biochemical markers (reactive oxygen species, malondialdehyde, antioxidant capacity and enzymes); E3, in vitro functional tests (cervical-mucus penetration, oocyte binding, in vitro fertilization); E4, in vivo fertility (pregnancy or lambing rate). [sic] denotes a unit reproduced exactly as printed in the original article after verification against it; such values may contain errors in the original and should be interpreted with caution.

**Table 2:** Amino acids in semen extenders.

Reference	Species and Sperm Source	Model	Supplement(s)	Concentration in Extender (as Reported)	Extender	Main Outcomes	Endpoint
Mahmood & Naoman [71]	Ram, epididymal	Chilled	Arginine, D-alanine, methionine	0.4 mM arginine, 15 mM D-alanine, 0.2 mM methionine	Sodium citrate, fructose, egg yolk	In total, 0.2 mM methionine showed the highest motility and lowest sperm abnormalities after 72 h of chilled storage, but arginine and alanine also improved sperm parameters compared to the control.	E1
Makarevich et al. [67]	Ram, ejaculated	Chilled	Glutathione	0.5, 1.5, and 5 mM	Tris-based egg yolk free	Glutathione had a positive effect on the total and progressive motility and membrane stability, while reducing the occurrence of apoptosis/necrosis for up to 48 h. No improvement in penetration or heterologous cleavage rate at 1.5 mM after 48 h.	E3
Shi et al. [66]	Ram, ejaculated	Chilled	Glutathione	50, 100 and 200 mM [sic]	Tris–egg yolk	In total, 200 mM slowed the decline of GLUT 3 and 8 transporter proteins. Both 100 mM and 200 mM showed improved total motility, membrane integrity, motion parameters, antioxidant capacity and mitochondrial activity at 72 h of storage. In total, 50 mM raised progressive motility and viability and lowered MDA but not total motility. Fertility not evaluated.	E2
Kafi et al. [63]	Ram, ejaculated	Cryopreserved	Cysteine	0.5 and 1.0 mg/mL	Tris–egg yolk	In total, 1.0 mg/mL cysteine was the most favorable when added pre-freeze and post-thaw, increasing motility, viability, and cervical mucus penetration and reducing oxidative stress.	E3
Toker & Dogan [11]	Ram, ejaculated	Cryopreserved	Methionine, cysteine	Methionine (2.5 and 5 mM), Cysteine (1 and 2 mM)	Tris–egg yolk	Both cysteine and methionine (both concentrations) were comparable at increasing sperm resilience parameters immediately post-thaw. In total, 2.5 mM methionine showed the highest motility and membrane integrity 6 h post-thaw, while acrosome and DNA integrity remained comparable across both groups and their concentrations.	E1
Salimi et al. [64]	Ram, ejaculated	Cryopreserved	Cysteamine, cysteamine loaded on selenium nanoparticles	1, 5, 25, and 125 µg/mL	Tris–egg yolk	In total, 1 µg/mL cysteamine loaded on selenium nanoparticles showed the most enhancement of sperm parameters (motility, viability, DNA and plasma membrane integrity, abnormality, and superoxide dismutase activity) and reduced malondialdehyde levels.	E2
Berean et al. [70]	Ram, ejaculated	Cryopreserved	Arginine	0.1 mM	Biladyl	No significant differences were found.	E4
Shahat et al. [62]	Ram, ejaculated	Cryopreserved	L-arginine	0.5 or 1 mM	Steridyl	Both concentrations improved motility: 1 mM showed the most improvement for sperm abnormalities and acrosome integrity, and 0.5 mM reduced DNA fragmentation the most. L-arginine 0.5 mM raised progressive motility from 26.1 to 34.2%; melatonin 1 mM raised total motility from 51.7 to 59.8%.	E2
Masoudi et al. [65]	Ram, ejaculated	Cryopreserved	Glutathione	0.5, 1, 2, 4, and 8 mM	Tris–soybean lecithin	Glutathione increased motility, antioxidant capacity, and viability and decreased acrosome damage with no significant additional effect on sperm cell morphology. In total, 8 mM glutathione increased lipid peroxidation and DNA fragmentation. Pregnancy did not differ from the control (20.0 vs. 30.0% with 4 mM).	E4
Hajihassani et al. [68]	Ram, epididymal	Cryopreserved	L-glutamine, glutathione	2.5 or 5 mM	Tris–egg yolk	L-glutamine had no effect, but glutathione at 5 mM improved motility parameters (48.7 to 61.6%) and improved in vitro fertilization outcomes post-thaw.	E3
de Souza et al. [72]	Ram, ejaculated	Cryopreserved	L-carnitine	5 and 10 mM	Tris–egg yolk or optiXcell	L-carnitine had no consistent beneficial effect at either concentration with either extender used.	E2
Kia & Vatankhah [73]	Ram, ejaculated	Cryopreserved	D-aspartic acid	5, 10, and 15 mg/L	Tris–soybean lecithin	In total, 10 mg/L was the most favorable among those tested, increasing total motility (47.5 to 62.2%), membrane integrity, mitochondrial activity and total antioxidant capacity while reducing malondialdehyde and abnormalities. In total, 5 mg/L gave smaller gains, and 15 mg/L did not differ from the control.	E2

E1, sperm-quality variables (motility, kinematics, viability, membrane, acrosome and mitochondrial integrity, DNA integrity); E2, oxidative and biochemical markers (reactive oxygen species, malondialdehyde, antioxidant capacity and enzymes); E3, in vitro functional tests (cervical-mucus penetration, oocyte binding, in vitro fertilization); E4, in vivo fertility (pregnancy or lambing rate). [sic] denotes a unit reproduced exactly as printed in the original article after verification against it; such values may contain errors in the original and should be interpreted with caution.

**Table 3:** Quinones and nitroxides in semen extenders.

Reference	Species and Sperm Source	Model	Supplement(s)	Concentration in Extender (as Reported)	Extender	Main Outcomes	Endpoint
Barfourooshi et al. [75]	Ram, ejaculated	Cryopreserved	MitoQ ^a^	1, 10, 100 and 1000 nM	Tris–soybean lecithin	In total, 10 and 100 nM MitoQ showed higher motility, viability and mitochondrial membrane potential, as well as reduced lipid peroxidation, ROS levels and DNA damage, and increased the pregnancy rate after insemination (33.3 to 52.2% with 100 nM).	E4
Masoudi et al. [76]	Ram, ejaculated	Cryopreserved	CoQ10 ^b^	1, 2, 5 and 10 μM	Tris–soybean lecithin or Tris–egg yolk	In total, 1 and 2 μM in both extenders showed greater motility, membrane integrity and mitochondrial membrane potential than other groups, while 2 μM in either extender exhibited the highest viability and lowest levels of lipid peroxidation and lowest number of apoptotic-like changes.	E2
Inanc et al. [77]	Ram, ejaculated	Cryopreserved	Thymoquinone	10, 25, 50, and 100 μg/mL	Tris–egg yolk	In total, 100 μg/mL thymoquinone increased total and progressive motility, and 50 μg/mL increased plasma membrane and acrosome integrity. All thymoquinone groups had less DNA damage than the control, and 50 and 100 μg/mL lowered mitochondrial ROS.	E2
Shi et al. [78]	Ram, ejaculated	Cryopreserved	Mito-TEMPO	20, 40 and 60 μM	Tris–egg yolk	In total, 40 μM of Mito-TEMPO showed the highest post-thaw motility and sperm kinematics, and it also resulted in a higher pregnancy rate and the upward regulation of 179 differentially expressed proteins.	E4
Zarei et al. [79]	Ram, ejaculated	Cryopreserved	Mito-TEMPO	0.5, 5, 50 and 500 µM	Tris–egg yolk, 200 IU/mL catalase	In total, 5 and 50 μM of Mito-TEMPO improved motility, viability, mitochondrial activity and membrane function, while reducing lipid peroxidation, ROS levels, and apoptotic-like changes. No effect on DNA fragmentation or morphology.	E2
Asadzadeh et al. [80]	Ram, ejaculated	Cryopreserved	Mito-TEMPO	0.5, 5, 50 and 500 µM	Tris–soybean lecithin, 200 IU/mL catalase	In total, 5 and 50 µM Mito-TEMPO showed higher motility, acrosome/membrane integrity, viability, mitochondrial membrane potential and higher pregnancy rates (25.3 to 44.0% with 50 µM), while reducing lipid peroxidation and apoptotic-like changes. All concentrations showed less DNA fragmentation but higher hydrogen peroxide levels.	E4

^a^ Mitoquinone; ^b^ Coenzyme Q10. E1, sperm-quality variables (motility, kinematics, viability, membrane, acrosome and mitochondrial integrity, DNA integrity); E2, oxidative and biochemical markers (reactive oxygen species, malondialdehyde, antioxidant capacity and enzymes); E3, in vitro functional tests (cervical-mucus penetration, oocyte binding, in vitro fertilization); E4, in vivo fertility (pregnancy or lambing rate).

**Table 4:** Flavonoid polyphenols in semen extenders.

Reference	Species and Sperm Source	Model	Supplement(s)	Concentration in Extender (as Reported)	Extender	Main Outcomes	Endpoint
Avdatek et al. [90]	Ram, ejaculated	Cryopreserved	Baicalein	0.5, 1, and 2 mM	Tris–egg yolk	In total, 0.5 mM baicalein preserved motility and had less chromatin damage than control and other concentrations. In total, 1 and 2 mM reduced motility (total motility 38.5 and 16.2% vs. 59.5% in the control). No concentration improved oxidative stress parameters.	E2
Khan et al. [88]	Ram, ejaculated	Cryopreserved	Quercetin	10 or 20 μM	Tris–egg yolk, 0, 1 or 2 mM ATP ^a^	In total, 1 mM ATP and 20 μM quercetin (separately) resulted in higher motility parameters, viability, and membrane integrity compared to all other groups. Both concentrations of quercetin alone improved motility parameters. In total, 1 mM ATP and 10 μM quercetin increased the pregnancy rate after laparoscopic insemination (27.5 to 58.1%).	E4
Najafi et al. [91]	Ram, epididymal	Cryopreserved	Rutin	0.5, 0.75, 1, and 1.25 mM	Tris–egg yolk	In total, 0.75 and 1 mM rutin significantly improved motility, mitochondrial activity and membrane integrity, increased ATP levels and antioxidant capacity, as well as reduced ROS and apoptosis.	E2
Yeni et al. [92]	Ram, ejaculated	Cryopreserved	Hesperidin	10, 50, 100, 250, and 500 µg/mL	Tris–egg yolk	No dose showed any effect on motility except 500 µg/mL, which lowered motility. Acrosome integrity and lipid peroxidation levels were improved with 10 µg/mL. In total, 10 and 50 µg/mL prevented chromatin damage.	E2
Arruda et al. [89]	Ram, ejaculated	Cryopreserved	Myricetin	20, 30, 40, 100, 200, 300, and 400 nM	Tris–egg yolk	No antioxidant effect or direct effect on motility parameters with any of the tested concentrations.	E2

^a^ Adenosine triphosphate. E1, sperm-quality variables (motility, kinematics, viability, membrane, acrosome and mitochondrial integrity, DNA integrity); E2, oxidative and biochemical markers (reactive oxygen species, malondialdehyde, antioxidant capacity and enzymes); E3, in vitro functional tests (cervical-mucus penetration, oocyte binding, in vitro fertilization); E4, in vivo fertility (pregnancy or lambing rate).

**Table 6:** Sources of mixed polyphenol supplements in semen extenders.

Reference	Species and Sperm Source	Model	Supplement(s)	Extraction	Concentration in Extender (as Reported)	Extender	Main Outcomes	Endpoint
Ali et al. [98]	Ram, ejaculated	Cryopreserved	*Basella rubra* fruit Extract	Fresh fruit juice, no solvent, and not standardized	0.6%, 0.8%, 1.6% (basis not stated)	Sodium citrate, glucose, egg yolk	Among the concentrations tested, 0.6% gave the most favorable results for sperm motility (71 vs. 53%) and membrane integrity. In total, 1.6% did not differ from the control. When pre-freeze values were accounted for, only 0.8% remained above the control.	E1
Khalil et al. [99]	Ram, ejaculated	Cryopreserved	Quinoa seed extract	Aqueous crude extract, partially standardized by batch characterization (total tannin, phenolic, and flavonoid content)	250, 500, 750 and 1000 µg/mL	Tris–soybean lecithin	Of all the concentrations tested, 750 and 1000 µg/mL gave the most favorable viability and motility results. In total, 500–1000 µg/mL reduced apoptotic-like changes. All concentrations improved total antioxidant capacity and reduced malondialdehyde and hydrogen peroxide levels by promoting antioxidant-related genes.	E2
Merati et al. [100]	Ram, epididymal	Cryopreserved	Ginger and echinacea extracts	Aqueous crude extract, partially standardized by batch characterization (total phenolic—both and saccharides—echinacea)	5, 10 and 20 mg/L	Tris, fructose, citric acid, egg yolk	In total, 10 mg/L ginger and 20 mg/L echinacea extracts provided the most favorable effects among those tested, including significantly lower malondialdehyde levels and higher sperm viability and membrane/acrosome integrity. Cleavage after in vitro fertilization was higher with 20 mg/L ginger and 10 mg/L echinacea.	E3
Najafi et al. [101]	Ram, ejaculated	Cryopreserved	Fennel extract	Aqueous crude extract, no standardization or characterization	5, 10 and 15 mg/L	Tris–soybean lecithin	In total, 10 mg/L fennel extract exhibited the most favorable results when added to sperm with a concentration of 200 × 10^6^ sperm/mL.	E2

Percentage concentrations are reproduced as reported; their basis is indicated only where specified in the original publication. E1, sperm-quality variables (motility, kinematics, viability, membrane, acrosome and mitochondrial integrity, DNA integrity); E2, oxidative and biochemical markers (reactive oxygen species, malondialdehyde, antioxidant capacity and enzymes); E3, in vitro functional tests (cervical-mucus penetration, oocyte binding, in vitro fertilization); E4, in vivo fertility (pregnancy or lambing rate).

**Table 7:** *Moringa oleifera* extracts in semen extenders.

Reference	Species and Sperm Source	Model	Supplement(s)	Extraction	Concentration in Extender (as Reported)	Extender	Main Outcomes	Endpoint
El-Seadawy et al. [18]	Ram, ejaculated	Cryopreserved	Moringa leaf methanolic extract	Methanolic leaf extract (30 g leaves/300 mL methanol), partially standardized (total phenolic and flavonoid content, antioxidant-capacity assays, and high-performance liquid chromatography profiling)	0.48, 0.56, and 0.64 mg/mL	Tris–egg yolk	In total, 0.56 and 0.64 mg/mL MLME ^a^ maintained motility, viability, membrane/acrosome integrity and reduced sperm abnormality, all concentrations lowered malondialdehyde levels and increased superoxide dismutase activity.	E2
Shokry et al. [17]	Ram, ejaculated	Cryopreserved	*Moringa oleifera* leaf extract	70% ethanolic leaf extract, partially characterized in authors’ previous study	300 and 600 mg/mL [sic]	Tris–egg yolk (BullXcell)	In total, 600 mg/mL [sic] MOLE ^b^ increased the pregnancy rate from 28.6 to 52.4% compared to the control group, both concentrations improved viability, membrane/acrosome integrity, and enzymatic antioxidant activity and reduced DNA damage.	E4
Carrera-Chávez et al. [104]	Ram, ejaculated	Cryopreserved	*Moringa oleifera* seed extract	80% (*v*/*v*) methanolic seed extract, partially characterized (total polyphenolic and flavonoid), no standardization	0.5, 5.0, and 10.0 mg/mL	Tris–egg yolk	No effect on acrosome integrity or mitochondrial activity: 0.5 and 5.0 mg/mL MOSE ^c^ increased antioxidant capacity, while 0.5 mg/mL decreased membrane damage and increased viability and progressive motility.	E1

^a^ *Moringa oleifera* leaf methanolic extract, ^b^ *Moringa oleifera* leaf extract, ^c^ *Moringa oleifera* seed extract. E1, sperm-quality variables (motility, kinematics, viability, membrane, acrosome and mitochondrial integrity, DNA integrity); E2, oxidative and biochemical markers (reactive oxygen species, malondialdehyde, antioxidant capacity and enzymes); E3, in vitro functional tests (cervical-mucus penetration, oocyte binding, in vitro fertilization); E4, in vivo fertility (pregnancy or lambing rate). [sic] denotes a unit reproduced exactly as printed in the original article after verification against it; such values may contain errors in the original and should be interpreted with caution.

**Table 8:** Hormones and growth factors in semen extenders.

Reference	Species and Sperm Source	Model	Supplement(s)	Concentration in Extender (as Reported)	Extender	Main Outcomes	Endpoint
Rateb et al. [110]	Ram, ejaculated	Chilled and Cryopreserved	Melatonin	0.3 mM	Tris–egg yolk	Melatonin preserved progressive motility and percentage of live and normal spermatozoa and slightly helped with antioxidant capacity for both chilled and cryopreserved samples.	E2
Carriço et al. [69]	Ram, ejaculated	Cryopreserved	Melatonin	1 mM	Tris–egg yolk, glutathione (5 mM; with and without)	Melatonin alone improved only progressive motility (20.5 to 26.7%) with no effect on total motility or viability; may provide protective effects when combined with glutathione in proper doses.	E1
Li et al. [111]	Ram, ejaculated	Cryopreserved	Melatonin	0.1, 0.5, 1.0, 1.5, and 2.0 mM	Optidyl	In total, 0.5 mM melatonin increased enzymatic antioxidant activity and improved motility parameters and both acrosome and plasma membrane integrity.	E2
Shahat et al. [62]	Ram, ejaculated	Cryopreserved	Melatonin	0.5 and 1 mM	Steridyl	Both concentrations improved motility: 1 mM showed the most improvement for sperm abnormalities and acrosome integrity, and 0.5 mM reduced DNA fragmentation the most. L-arginine 0.5 mM raised progressive motility from 26.1 to 34.2%; melatonin 1 mM raised total motility from 51.7 to 59.8%.	E2
Fang et al. [20]	Ram, ejaculated	Cryopreserved	Melatonin	0.1 μM	Glucose–egg yolk	Melatonin suppressed mitochondrial permeability transition pore (mPTP) opening, protected mitochondria, and improved sperm quality and pregnancy rate (16.0 to 34.8%).	E4
Sapanidou et al. [112] ^‡^	Bull, ejaculated	Cryopreserved (post-thaw incubation)	Erythropoietin	10 and 100 mIU/μL	Sperm TALP (after thawing)	Moderate effect on superoxide anion levels, but at 100 mIU/μL total antioxidant capacity was increased and the proportion of rapid sperm at 240 min (21.7 to 37.2%). In total, 100 mIU/μL acted as a capacitation factor.	E2
Pinto et al. [113] ^‡^	Bull, ejaculated	Cryopreserved	Insulin-like growth factor 1 (IGF-1), insulin	100 and 200 ng/mL (IGF-1), 150 and 200 µUI/mL (insulin) [sic]	Botubov or Triladyl	IGF-1 showed no effect at either concentration; 150 µUI/mL insulin [sic] improved preservation of motility, plasma membrane integrity, and mitochondrial membrane potential in both extenders.	E1
Oliveira Resende et al. [114] ^‡^	Boar, ejaculated	Cryopreserved and Chilled	IGF-1, anti-IGF-1	30 ng/mL (IGF-1) and 60 ng/mL (anti-IGF-1)	Beltsville Thawing Solution	Antioxidant potential was improved with both IGF-1 and anti-IGF-1 when combined with glutathione, but these combinations did decrease acrosomal integrity; IGF-1 and anti-IGF-1 both maintained sperm quality for chilled and cryopreserved sperm.	E2

^‡^ Denotes a comparative study. E1, sperm-quality variables (motility, kinematics, viability, membrane, acrosome and mitochondrial integrity, DNA integrity); E2, oxidative and biochemical markers (reactive oxygen species, malondialdehyde, antioxidant capacity and enzymes); E3, in vitro functional tests (cervical-mucus penetration, oocyte binding, in vitro fertilization); E4, in vivo fertility (pregnancy or lambing rate). [sic] denotes a unit reproduced exactly as printed in the original article after verification against it; such values may contain errors in the original and should be interpreted with caution.

**Table 9:** Fat supplements in semen extenders.

Reference	Species and Sperm Source	Model	Supplement(s)	Concentration in Extender (as Reported)	Extender	Main Outcomes	Endpoint
Soltani [120]	Ram, ejaculated	Cryopreserved	OA ^f^	0.5 and 1 mM	Tris-based, trehalose (25, 50 mM)	In total, 1 mM OA + 25 mM trehalose significantly improved the total motility and showed increased superoxide dismutase levels compared to other concentrations; all concentrations of OA + trehalose reduced levels of MDA ^g^.	E2
Mortazavi et al. [122]	Ram, ejaculated	Cryopreserved	LL-OA ^a^, CD-OA ^b^	0.25, 0.50, 1.0 and 2.0 mM (LL-OA) and 0, 0.25, 0.50, 1 and 2 mM (CD-OA)	Tris, citric acid, fructose and glycerol	LL-OA or CD-OA at 0.25 and 0.50 mM had highest resistance to thermal shock and freezing.	E2
Inanc et al. [123]	Ram, ejaculated	Cryopreserved	CLC ^c^ and 7-DCLC ^d^	1.5, 2.5, or 3.5 mg/120 × 10^6^ sperm	Tris, citric acid, glucose, glycerol, egg yolk, and trehalose	CLC + trehalose showed significant improvements in motility, plasma membrane integrity, and mitochondrial membrane potential, with 2.5 mg having the most favorable results among those tested.	E1
Mafolo et al. [124]	Ram, ejaculated	Cryopreserved	Phosphatidylcholine (PC ^h^)	0.25, 0.50, and 0.75 mg/mL	Tris-based extender (1:2) and egg yolk (with and without)	PC with and without egg yolk showed no improvement; samples with egg yolk had higher viability and motility.	E1
Towhidi et al. [14] ^†^	Ram, ejaculated	Cryopreserved	n-3 fatty acids (Viva Pharmaceutical Inc., Richmond, BC, Canada)	0.1, 1, 10 ng/mL	Andromed and α-tocopherol (0, 0.1, 0.2 mM)	Progressive motility improved (29.0 to 32.5%) with 1 ng/mL + 0.1 mM α-tocopherol, total motility unchanged, increased DHA ^e^ levels pre-freeze.	E1
Bucak et al. [121] ^‡^	Goat, ejaculated	Cryopreserved	Lipid mixtures (from a liposome kit)	321.99, 841.33 μg/mL	Tris, trizma, fructose, citric acid, glycerol, egg yolk, melatonin (0.25, 1.0 mM)	Low-dose lipid mixtures alone or in combination with melatonin significantly preserve sperm motility, membrane/acrosome integrity and DNA.	E1

^a^ Liposome-loaded oleic acid, ^b^ cyclodextrin-loaded oleic acid, ^c^ cyclodextrin-loaded cholesterol, ^d^ 7-dehydrocholesterol loaded cyclodextrin, ^e^ docosahexaenoic acid, ^f^ oleic acid, ^g^ malondialdehyde, ^h^ phosphatidylcholine. ^†^ Denotes background/landmark study. ^‡^ Denotes a comparative study. E1, sperm-quality variables (motility, kinematics, viability, membrane, acrosome and mitochondrial integrity, DNA integrity); E2, oxidative and biochemical markers (reactive oxygen species, malondialdehyde, antioxidant capacity and enzymes); E3, in vitro functional tests (cervical-mucus penetration, oocyte binding, in vitro fertilization); E4, in vivo fertility (pregnancy or lambing rate).

**Table 10:** Saccharides in semen extenders.

Reference	Species and Sperm Source	Model	Supplement(s)	Concentration in Extender (as Reported)	Extender	Main Outcomes	Endpoint
Zhao et al. [128]	Ram, ejaculated	Chilled (0 °C)	Trehalose	5, 10, 15, 20 mM	Tris–fructose–egg yolk	At 0 °C, 5 mM trehalose preserved acrosome integrity better than the unsupplemented control, but it did not differ significantly from the other trehalose concentrations. Pregnancy and lambing rates did not differ significantly between 5 mM and control.	E4
Özmen et al. [129]	Ram, ejaculated	Cryopreserved	Trehalose	12.5, 25, and 50 mmol/L	Tris egg yolk, iodixanol (0, 1.25, 2.5, 5%), glycerol	Trehalose (25 or 50 mmol/L) or iodixanol (5%) separately helped protect functional and morphological integrity at 1 and 4 h, trehalose (25 mmol/L) and iodixanol (5%) significantly improved post-thaw longevity.	E1
Ahmed et al. [21]	Ram, epididymal	Cryopreserved	Glucose, fructose	1.25% (*w*/*v*)	Tris–citric acid or sodium citrate, egg yolk, glycerol	Fructose in a Tris–citric acid extender maintained the highest sperm quality, but using fructose with a sodium citrate extender also has potential.	E1
Herdis et al. [130]	Ram, ejaculated	Cryopreserved	Sucrose	0.2, 0.4, 0.6 g/100 mL	Tris extender	In total, 0.2 g/100 mL sucrose improved viability (52.7% to 62.8%) and membrane integrity. Motility improved only at 0.4–0.6 g/100 mL.	E1
Shehab-El-Deen et al. [131]	Ram, ejaculated	Cryopreserved	Gum arabic	5%	Tris (with/without egg yolk) supplemented with cysteine or ascorbic acid	Gum arabic supplementation provided similar results to those of egg yolk. In total, 5% gum arabic was the most favorable when supplemented with 0.5 or 1 mM cysteine or 0.5 mM ascorbic acid.	E1
Khanzadeh et al. [132]	Ram, ejaculated	Cryopreserved	Apricot tree gum (3% (*w*/*v*) apricot gum with water; chemical components classified)	10, 15 and 20 mg/mL	Tris, fructose, citric acid monohydrate, egg yolk, glycerol	All three doses improved compared to the control and did not differ between each other, but 20 mg/mL provided the highest total motility and membrane viability and integrity while reducing malondialdehyde the most, 10 and 15 mg/mL had the highest progressive motility.	E2
Gungor et al. [133] ^†^	Ram, ejaculated	Cryopreserved	Trehalose	25, 50 mM	Tris, fructose, citric acid, egg yolk, glycerol, Catalase (with and without)	In total, 50 mM trehalose alone or with 400 μg catalase showed positive effects on sperm parameters.	E1
Wu et al. [126] ^†^	Ram, ejaculated	Cryopreserved	Mannitol, sorbitol, xylitol, glucose, fructose	55.5 mM	Tris, citric acid monohydrate, egg yolk, glycerol	Mannitol and sorbitol showed better results in all motility parameters (except linear velocity) as well as acrosome and membrane integrity than glucose and fructose.	E1
Nurlatifah et al. [127] ^‡^	Goat, ejaculated	Cryopreserved	Dextrose, sucrose, raffinose	0.6%	Tris egg yolk	Sucrose was more effective at maintaining sperm parameters than dextrose and raffinose.	E1

^‡^ Denotes a comparative study. ^†^ Denotes background/landmark study. Percentage concentrations are reproduced as reported; their basis is indicated only where specified in the original publication. E1, sperm-quality variables (motility, kinematics, viability, membrane, acrosome and mitochondrial integrity, DNA integrity); E2, oxidative and biochemical markers (reactive oxygen species, malondialdehyde, antioxidant capacity and enzymes); E3, in vitro functional tests (cervical-mucus penetration, oocyte binding, in vitro fertilization); E4, in vivo fertility (pregnancy or lambing rate).

## Data Availability

No new data were created or analyzed in this study. Data sharing is not applicable to this article.

## Supplementary Materials

The following supporting information can be downloaded at https://www.mdpi.com/article/10.3390/ani16203305/s1, Table S1: Appraisal of methodological and reporting quality of the 67 studies included in the review (64 evidence-set studies and 3 background studies), using a seven-item checklist adapted from the SYRCLE risk-of-bias tool and the ARRIVE 2.0 guidelines.
